# Supplementary material for: Dynamics of sputum conversion during effective tuberculosis treatment: A systematic review and meta-analysis
Source: PLoS Med. 2021 Apr 26;18(4):e1003566. doi: 10.1371/journal.pmed.1003566 (PMC8109831; doi:10.1371/journal.pmed.1003566)
Supplement: S1 Appendix — (DOCX) [file pmed.1003566.s002.docx]

**S2 Appendix: Search update**

At the request of the editors and in order to assess whether any studies published subsequent to the date of our searches would change the review’s findings, we conducted a literature search of OvidSP Medline and EMBASE on 22^nd^ November 2020. For this we ran both full systematic searches, as detailed in S1 Table and S2 Table, followed by addition of terms identified as being common as either keywords or in title/abstracts of studies included in the review (Table A).

**Table A: Search strategy employed for review of search results.**

Databases: OvidSP Medline and EMBASE. Date of search 22^nd^ November 2020.

Search 1 and RCT search are saved searches corresponding to the searches detailed in S1 Table and S2 Table.

|  | **Terms** | **N records** |
| --- | --- | --- |
| 1 | Sputum/ | 21,558 |
| 2 | conver*.mp. | 517,435 |
| 3 | conver*.ti,ab. | 494,463 |
| 4 | sputum.ti,ab. | 27,787 |
| 5 | smear.mp. | 28,062 |
| 6 | smear.ti,ab. | 27,382 |
| 7 | culture.ti,ab. | 529,455 |
| 8 | or (1-7) | 1,080,651 |
| 9 | ([search 1] or [RCT search]) and 8 | 843 |
| 10 | Limit 8 to yr="2018 -Current" | 105 |

Of 105 studies, four met the inclusion criteria for the study, as detailed in Table B. The median study size was 88 participants (range 60-103). None included evaluation of sputum dynamics during TB treatment as a stated objective. Three studies were phase 2 randomised controlled trials which reported data from a standard of care arm, one was a retrospective cohort study. Three reported the proportion converted at two months of TB treatment, the timepoint for which the most data exist from our systematic review; one additionally reported time to liquid culture conversion. The fourth study reported time to smear negativity. None provided data stratified by subgroups of interest, such as HIV, smear grading or presence of absence of cavitation on chest radiograph. The estimates presented were consistent with those from studies included in the systematic review.

**Table B: Details of eligible studies.**

|  | Type | Number with outcome | Outcome reported | Subgroup data reported? |
| --- | --- | --- | --- | --- |
| Al-Shaer [1] | Cohort (retrospective) | 103 | Time to smear negativity (weekly smear) | Fixed dose combinations vs single tablets |
| Tweed [2] | Phase 2 randomised controlled trial | 59 | Time to liquid culture conversion and proportion converted at 2 months | No |
| Velasquez [3] | Phase 2 randomised controlled trial | 60 | Proportion converted at 2 months | No |
| Lee [4] | Phase 2 randomised controlled trial | 127 | Proportion converted at 2 months | No |

This literature search attempted to establish whether it was likely that a significant body of work had been published subsequent to the date of the previous systematic searches, materially impacting on our understanding of the dynamics of sputum bacteriological clearance during TB treatment. Inclusion of the identified studies would not change the results or conclusions of this review. Whilst it is possible that this limited search may have missed some relevant studies, we found no published work which aimed to investigate sputum bacteriological clearance during TB treatment, or which addresses the gaps identified by the systematic review.

**References**

1. Al-Shaer MH, Elewa H, Alkabab Y, Nazer LH, Heysell SK. Fixed-dose combination associated with faster time to smear conversion compared to separate tablets of anti-tuberculosis drugs in patients with poorly controlled diabetes and pulmonary tuberculosis in Qatar. BMC Infect Dis. 2018. doi:10.1186/s12879-018-3309-0

2. Tweed CD, Dawson R, Burger DA, Conradie A, Crook AM, Mendel CM, et al. Bedaquiline, moxifloxacin, pretomanid, and pyrazinamide during the first 8 weeks of treatment of patients with drug-susceptible or drug-resistant pulmonary tuberculosis: a multicentre, open-label, partially randomised, phase 2b trial. Lancet Respir Med. 2019. doi:10.1016/S2213-2600(19)30366-2

3. Velásquez GE, Brooks MB, Coit JM, Pertinez H, Vásquez DV, Garavito ES, et al. Efficacy and safety of high-dose rifampin in pulmonary tuberculosis a randomized controlled trial. Am J Respir Crit Care Med. 2018. doi:10.1164/rccm.201712-2524OC

4. Lee JK, Lee JY, Kim DK, Yoon H Il, Jeong I, Heo EY, et al. Substitution of ethambutol with linezolid during the intensive phase of treatment of pulmonary tuberculosis: a prospective, multicentre, randomised, open-label, phase 2 trial. Lancet Infect Dis. 2019. doi:10.1016/S1473-3099(18)30480-8
