# Supplementary material for: Dynamics of sputum conversion during effective tuberculosis treatment: A systematic review and meta-analysis
Source: PLoS Med. 2021 Apr 26;18(4):e1003566. doi: 10.1371/journal.pmed.1003566 (PMC8109831; doi:10.1371/journal.pmed.1003566)
Supplement: S2 Appendix — (DOCX) [file pmed.1003566.s003.docx]

**Narrative review of animal studies**

The key data from each of the four publications that relates to infectiousness of patients with TB (to guinea pigs) and TB treatment, are reported here. The diverse methodologies and analytical approaches preclude data aggregation.

###### ***Riley, Am Rev Respir Dis (1962)***

From 1959–1961 monthly tuberculin skin testing (TST) was performed on 120 guinea pigs (approximately 2,880 TSTs) that were breathing air exhausted from a purpose-designed six-bedded isolation unit [1]***.*** Of all those admitted through the unit during this period, 107 patients, representing 61% of total bed-days, were identified as having culture-positive PTB. Some patients were not treated, though it is not clear whether this was for the entire period of their admission. It is stated that “*Treated patients were admitted to the ward at the time treatment was initiated*”.

In total 63 guinea pigs were defined as having been infected (TST conversion), from 51 of which a positive *M. tuberculosis* culture was obtained. 50 of these isolates were matched to an index patient on the basis of drug susceptibility profile and temporal relationship; 15 of these matched to a single patient with laryngeal TB. The number of guinea-pigs infected by patients with drug-susceptible TB is shown in Table A.

| **Table A. Number of guinea pigs infected by patients with drug-susceptible TB (Source: Riley *et al.* 1962)** [1] | | | | |
| --- | --- | --- | --- | --- |
| **Category** | | **Number of patients** | **Percentage of total patient-days** | **Number of infected guinea pigs** |
| Untreated patients | Non-infectors | 53 | 6% | 0 |
|  | Infectors | 8 | 1% | 29 |
| Treated patients | Non-infectors | 28 | 14.7% | 0 |
|  | Infectors | 1 | 0.3% | 1 |

As the authors state “*Evaluation of the effects of drug therapy on the transmission of tuberculosis is beset with a number of difficulties. Only 8 of 61 patients in the “untreated, susceptible organism” category infected any guinea pigs at all…and the patient with tuberculous laryngitis infected half of the total by himself (15 of 29)*”.

The presented data suggest that patients on treatment are less able to infect guinea pigs than patients not receiving treatment. However this study provides no data to inform the question of the timing of when this reduction in/interruption of infectiousness occurs.

##### ***Escombe, Clin Infect Dis (2007), PloS Med (2008)***

The experiments reported in these two papers describe the findings in a guinea pig population exposed to 66 people with HIV (not receiving antiretroviral therapy) with culture-positive PTB [2,3]. Twelve (or possibly 11) of the 66 patients transmitted *M. tuberculosis* to the guinea pigs, with 108 of the 135 guinea pig infections attributed to a single individual patient with AFB 3+ smear-positive multidrug-resistant TB who spent 11 of 32 ward-days awaiting initiation of second line anti-TB therapy. Though 38% of the inpatient bed-days were accounted for by patients with drug-susceptible disease, only 2% of guinea pig infections were drug-susceptible. Treatment is described as “sub-optimal” for 298/1790 (35%) patient bed-days (and “optimal” for 65%), though these categories are not clearly defined.

Figure A summarizes the entirety of the data presented; it is not possible to disentangle which patients infected which guinea pigs and when. Most patients linked to a transmission event had periods both on and off treatment so it is not posssible to attribute transmission to an on-treatment or off-treatment moment. There are no data to inform the question of the speed of the effect of treatment upon infectiousness from time of initiation.

| **Figure A. Summary of data from Escombe 2007 and 2008** [2,3] |
| --- |
| 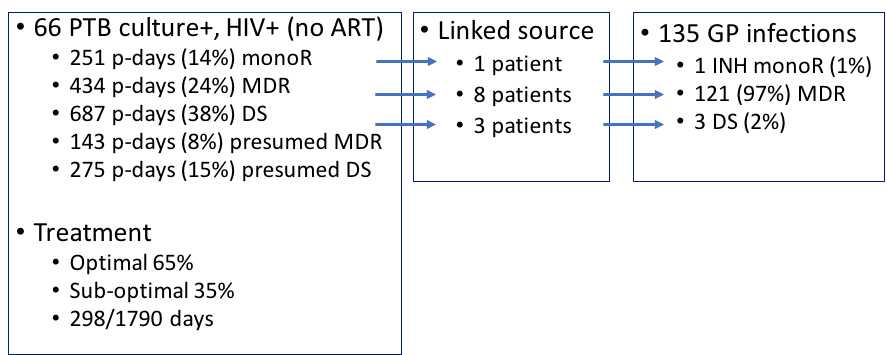 |
| **Abbreviations:**  ART: = antiretroviral therapy;  Culture+ = culture positive  DS = drug-susceptible;  GP = guinea pig;  HIV+ = HIV-positive;  INH = isoniazid;  MDR = multidrug-resistant  monoR = mono-resistance  PTB: pulmonary tuberculosis |

##### ***Dharmadhikari, Int J Tuberc Lung Dis (2014)***

In this paper the data from five earlier experiments are scrutinised with the specific purpose of exploring the effectiveness of TB treatment upon infectiousness [4]. Studies 1–4 were all intervention studies in which separated groups of guinea pigs were exposed either to air exhausted from isolation rooms where an intervention was being applied (either to the patient or the room or the air) or to control air which was exhausted from non-intervention rooms; only the control arm data are included in the analysis in this paper. The data reported are presented in Table B which combines data from the text and several tables.

| **Table B. Summary of data presented in Dharmadhikari 2014** [4] |
| --- |
| 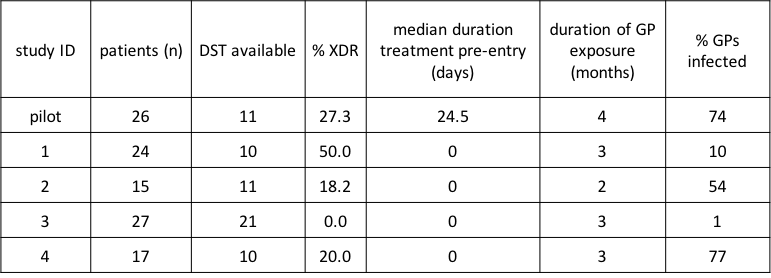 |
| **Abbreviations:**  DST = drug-susceptibility testing;  GP = guinea pig;  XDR = extensively drug-resistant |

The authors indicate that study groups with a higher proportion of patients subsequently identified as having had XDR-TB (which was unrecognised and thus inadequately treated at the time) were associated with a higher percentage of *Mtb* infections amongst the guinea pigs. However TB culture from guinea pigs was only performed in the pilot study and drug susceptibility testing was only available for 13 of 216 guinea pig infections (5% of total, 18% of pilot study), all of which were XDR. Attribution of guinea pig infection to periods on or off effective treatment is not possible from the available data. The speed of the effect of treatment cannot be elicited.

**References**

1. Riley RL, Mills CC, O’Grady F, Sultan LU, Wittstadt F, Shivpuri DN. Infectiousness of air from a tuberculosis ward. Ultraviolet irradiation of infected air: comparative infectiousness of different patients. Am Rev Respir Dis. 1962. doi:10.1164/arrd.1962.85.4.511

2. Escombe AR, Oeser C, Gilman RH, Navincopa M, Ticona E, Martinez C, et al. The Detection of Airborne Transmission of Tuberculosis from HIV-Infected Patients, Using an In Vivo Air Sampling Model. Clin Infect Dis. 2007. doi:10.1086/515397

3. Escombe AR, Moore DAJ, Gilman RH, Pan W, Navincopa M, Ticona E, et al. The infectiousness of tuberculosis patients coinfected with HIV. PLoS Med. 2008. doi:10.1371/journal.pmed.0050188

4. Dharmadhikari AS, Mphahlele M, Venter K, Stoltz A, Mathebula R, Masotla T, et al. Rapid impact of effective treatment on transmission of multidrug-resistant tuberculosis. Int J Tuberc Lung Dis. 2014. doi:10.5588/ijtld.13.0834
