# Supplementary material for: Dynamics of sputum conversion during effective tuberculosis treatment: A systematic review and meta-analysis
Source: PLoS Med. 2021 Apr 26;18(4):e1003566. doi: 10.1371/journal.pmed.1003566 (PMC8109831; doi:10.1371/journal.pmed.1003566)
Supplement: S1 Table — (DOCX) [file pmed.1003566.s006.docx]

| S1 Table. Details of databases searched and terms used for original search; run on December 1^st^ 2017 | | | | | | | |
| --- | --- | --- | --- | --- | --- | --- | --- |
| Ovid MEDLINE In-Process & Other Non-Indexed Citations, Ovid MEDLINE Daily and Ovid MEDLINE  Date: 1946 to 2017 November 30 | | OvidSP Embase Classic+Embase  Date: 1947 to 2017 November 30 | | OvidSP Global Health  Date: 1910 to 2017 Week 47 | | EBSCO CINAHL Plus Date: no limits | |
| 1 | carrier state/ (21,766) | 1 | infectiousness.ti,ab. (1,589) | 1 | infectivity/ (9,630) | S1 | (TI infectiousness) OR (AB infectiousness) (161) |
| 2 | infectiousness.ti,ab. (1,309) | 2 | infectivity.ti,ab. (29,264) | 2 | infectiousness.ti,ab. (883) | S2 | (TI infectivity) OR (AB infectivity) (506) |
| 3 | infectivity.ti,ab. (25,226) | 3 | ((transmis* or transmit*) adj2 (dynamic* or risk* or event? Or reservoir? Or less or more or remain or persist* or sustain* or probabilit*)).ti,ab. (18,979) | 3 | infectivity.ti,ab. (12,854) | S3 | (TI ((transmis* OR transmit*) N2 (dynamic* OR risk* OR event OR events OR 4reservoir* OR less OR more OR remain OR persist* OR sustain* OR panishse*))) OR (AB ((transmis* OR transmit*) N2 (dynamic* OR risk* OR event OR events OR reservoir* OR less OR more OR remain OR persist* OR sustain* OR probabilit*))) (4,458) |
| 4 | ((transmis* or transmit*) adj2 (dynamic* or risk* or event? or reservoir? or less or more or remain or persist* or sustain* or probabilit*)).ti,ab. (16,340) | 4 | (infection? Adj2 (reservoir? Or less or more or remain or persist* or sustain*)).ti,ab. (38,274) | 4 | ((transmis* or transmit*) adj2 (dynamic* or risk* or event? Or reservoir? Or less or more or remain or persist* or sustain* or probabilit*)).ti,ab. (8,784) | S4 | (TI (infection* N2 (reservoir* OR less OR more OR remain OR persist* OR sustain*))) OR (AB (infection* N2 (reservoir* OR less OR more OR remain OR persist* OR sustain*))) (4,651) |
| 5 | (infection? adj2 (reservoir? or less or more or remain or persist* or sustain*)).ti,ab. (30,172) | 5 | ((viable or live or infectious) adj2 (pathogen? Or bacteria or bacterium)).ti,ab. (8,420) | 5 | (infection? Adj2 (reservoir? Or less or more or remain or persist* or sustain*)).ti,ab. (14,299) | S5 | (TI ((viable OR live OR infectious) N2 (pathogen* OR bacteria OR bacterium))) OR (AB ((viable OR live OR infectious) N2 (pathogen* OR bacteria OR bacterium))) (575) |
| 6 | ((viable or live or infectious) adj2 (pathogen? or bacteria or bacterium)).ti,ab. (7,132) | 6 | or/1-5 (94,736) | 6 | ((viable or live or infectious) adj2 (pathogen? Or bacteria or bacterium)).ti,ab. (2,522) | S6 | S1 OR S2 OR S3 OR S4 OR S5 (10,087) |
| 7 | or/1-6 (99,604) | 7 | exp tuberculosis/ (242,865) | 7 | or/1-6 (39,977) | S7 | (MH “Tuberculosis+”) (17,381) |
| 8 | exp tuberculosis/ (190,560) | 8 | exp mycobacterium tuberculosis complex/ (77,788) | 8 | exp mycobacterium tuberculosis/ (73,017) | S8 | (MH “Mycobacterium Tuberculosis”) (2,797) |
| 9 | mycobacterium tuberculosis/ (47,471) | 9 | tuberculosis control/ (6,263) | 9 | tuberculosis.ti,ab. (71,344) | S9 | (TI tuberculosis) OR (AB tuberculosis) (15,336) |
| 10 | tuberculosis.ti,ab. (176,160) | 10 | tuberculosis.ti,ab. (206,771) | 10 | tb.ti,ab. (21,195) | S10 | (TI tuberculin) OR (AB tuberculin) (1,080) |
| 11 | tb.ti,ab. (43,071) | 11 | tb.ti,ab. (79,301) | 11 | or/8-10 (80,137) | S11 | (TI tb) OR (AB tb) (6,747) |
| 12 | or/8-11 (256,157) | 12 | or/7-11 (327,882) | 12 | 7 and 11 (1,477) | S12 | S7 OR S8 OR S9 OR S10 OR S11 (22,540) |
| 13 | 7 and 12 (2,452) | 13 | 6 and 12 (3,063) | 13 | (chinese or english or french or japanese or portuguese or russian or spanish).lg. (2,988,743) | S13 | S6 AND S12 (368) |
| 14 | (chinese or english or french or japanese or portuguese or russian or spanish).lg. (26,990,357) | 14 | (chinese or english or french or japanese or portuguese or russian or spanish). .lg. (31,240,565) | 14 | 12 and 13 (1,106) | S14 | S13 Limiters – Language: Chinese, English, French, Japanese, Portuguese, Spanish (367) |
| 15 | 13 and 14 (2,332) | 15 | 13 and 14 (2,913) | 15 | remove duplicates from 14 (1,105) |  |  |
| 16 | remove duplicates from 15 (2,162) | 16 | remove duplicates from 15 (2,761) |  |  |  |  |
