# Supplementary material for: Dynamics of sputum conversion during effective tuberculosis treatment: A systematic review and meta-analysis
Source: PLoS Med. 2021 Apr 26;18(4):e1003566. doi: 10.1371/journal.pmed.1003566 (PMC8109831; doi:10.1371/journal.pmed.1003566)
Supplement: S2 Table — (DOCX) [file pmed.1003566.s007.docx]

| S2 Table. Details of databases searched and terms used for secondary search; run on February 20^th^ 2018 | | | | | | | |
| --- | --- | --- | --- | --- | --- | --- | --- |
| Ovid MEDLINE In-Process & Other Non-Indexed Citations, Ovid MEDLINE Daily and Ovid MEDLINE  Date: 1946 to 2018 February 16 | | OvidSP Embase  Date: 1974 to 2018 February 16 | | OvidSP Global Health  Date: 1910 to 2018 Week 06 | | EBSCO CINAHL Plus Date: full database searched | |
| 1 | exp tuberculosis, pulmonary/ (72,595) | 1 | lung tuberculosis/ (61,525) | 1 | exp tuberculosis/ (69,715) | S1 | (MH “Tuberculosis, Pulmonary”) (4,148) |
| 2 | tuberculosis/ (99,245) | 2 | tuberculosis/ (115,121) | 2 | tuberculosis.ti,ab. (71,916) | S2 | (MH “Tuberculosis”) (12,326) |
| 3 | exp Tuberculosis, Multidrug-Resistant/ (6,669) | 3 | exp drug resistant tuberculosis/ (7,333) | 3 | tb.ti,ab. (21,579) | S3 | (MH “Tuberculosis, Multidrug-Resistant”) (1,703) |
| 4 | tuberculosis.ti,ab. (169,241) | 4 | tuberculosis.ti,ab. (177,224) | 4 | mdr-tb.ti,ab. (2,220) | S4 | (TI tuberculosis) OR (AB tuberculosis) (15,677) |
| 5 | tb.ti,ab. (40,846) | 5 | tb.ti,ab. (53,718) | 5 | xdr-tb.ti,ab. (570) | S5 | (TI tb) OR (AB tb) [6,927] |
| 6 | mdr-tb.ti,ab. (2,954) | 6 | mdr-tb.ti,ab. (4,118) | 6 | or/1-5 (80,272) | S6 | (TI “mdr-tb”) OR (AB “mdr-tb”) (479) |
| 7 | xdr-tb.ti,ab. (853) | 7 | xdr-tb.ti,ab. (1,257) | 7 | randomized controlled trials/ (29,327) | S7 | (TI “xdr-tb”) OR (AB “xdr-tb”) (127) |
| 8 | or/1-7 (226,367) | 8 | or/1-7 (243,269) | 8 | randomized.ab. (63,103) | S8 | S1 OR S2 OR S3 OR S4 OR S5 OR S6 OR S7 (22,456) |
| 9 | randomized controlled trial.pt. (453,810) | 9 | randomized controlled trial/ (487,494) | 9 | placebo.ab. (31,442) | S9 | (MH “Randomized Controlled Trials”) (66,801) |
| 10 | controlled clinical trial.pt. (92,162) | 10 | exp “controlled clinical trial (topic)”/ (145,459) | 10 | randomly.ab. (75,022) | S10 | (AB randomized) (110,522) |
| 11 | randomized.ab. (393,756) | 11 | randomized.ab. (569,353) | 11 | trial.ti. (27,552) | S11 | (AB placebo) (39,335) |
| 12 | placebo.ab. (183,608) | 12 | placebo.ab. (258,930) | 12 | or/7-11 (155,921) | S12 | (AB randomly) (60,865) |
| 13 | clinical trials as topic.sh. (182,599) | 13 | randomly.ab. (369,989) | 13 | ethambutol/ (2,321) | S13 | (TI trial) (77,531) |
| 14 | randomly.ab. (279,733) | 14 | trial.ti. (242,339) | 14 | isoniazid/ (7,542) | S14 | S9 OR S10 OR S11 OR S12 OR S13 (236,660) |
| 15 | trial.ti. (174,081) | 15 | or/9-14 (1,324,245) | 15 | pyrazinamide/ (1,516) | S15 | (MH “Isoniazid”) (829) |
| 16 | or/9-15 (1,116,904) | 16 | ethambutol/ (27,323) | 16 | rifampicin/ (8,560) | S16 | (MH “Rifampin”) (1,264) |
| 17 | exp animals/ not humans.sh. (4,426,664) | 17 | isoniazid/ (53,149) | 17 | isoniazid.ti,ab. (8,197) | S17 | (MH “Ethambutol”) [9] |
| 18 | 16 not 17 (1,027,253) | 18 | pyrazinamide/ (21,643) | 18 | isonicotinic acid hydrazide.ti,ab. (263) | S18 | (MH “Pyrazinamide”) [215] |
| 19 | Isoniazid/ (17,851) | 19 | rifampicin/ (81,004) | 19 | phthivazid.ti,ab. (4) | S19 | (TI isoniazid) OR (AB isoniazid) [949] |
| 20 | isoniazid.ti,ab. (14,060) | 20 | aminosalicylic acid plus isoniazid/ (5) | 20 | phthivazide.ti,ab. (5) | S20 | (TI “isonicotinic acid hydrazide”) OR (AB “isonicotinic acid hydrazide”) [5] |
| 21 | isonicotinic acid hydrazide.ti,ab. (1,901) | 21 | cycloserine plus isoniazid/ (0) | 21 | tubazide.ti,ab. (4) | S21 | (TI phthivazid) OR (AB phthivazid) [0] |
| 22 | phthivazid.ti,ab. (77) | 22 | ethambutol plus isoniazid/ (84) | 22 | rifampin.ti,ab. (3,016) | S22 | (TI phthivazide) OR (AB phthivazide) [0] |
| 23 | phthivazide.ti,ab. (64) | 23 | isoniazid plus rifampicin/ (1,009) | 23 | benemycin.ti,ab. (3) | S23 | (TI tubazide) OR (AB tubazide) [0] |
| 24 | tubazide.ti,ab. (16) | 24 | rifampicin plus trimethoprim/ (14) | 24 | rifadin.ti,ab. (9) | S24 | (TI rifampin) OR (AB rifampin) [692] |
| 25 | Rifampin/ (16,499) | 25 | isoniazid plus pyrazinamide plus rifampicin/ (306) | 25 | rifampicin.ti,ab. (7,340) | S25 | (TI benemycin) OR (AB benemycin) [0] |
| 26 | rifampin.ti,ab. (7,525) | 26 | ethambutol plus isoniazid plus pyrazinamide plus rifampicin/ (479) | 26 | rimactan.ti,ab. (5) | S26 | (TI rifadin) OR (AB rifadin) [1] |
| 27 | benemycin.ti,ab. (16) | 27 | isoniazid.ti,ab. (17,268) | 27 | rimactane.ti,ab. (7) | S27 | (TI rifampicin) OR (AB rifampicin) [849] |
| 28 | rifadin.ti,ab. (46) | 28 | isonicotinic acid hydrazide.ti,ab. (1,720) | 28 | ethambutol.ti,ab. (2,418) | S28 | (TI rimactan) OR (AB rimactan) [0] |
| 29 | rifampicin.ti,ab. (14,105) | 29 | phthivazid.ti,ab. (38) | 29 | myambutol.ti,ab. (8) | S29 | (TI rimactane) OR (AB rimactane) [1] |
| 30 | rimactan.ti,ab. (26) | 30 | phthivazide.ti,ab. (47) | 30 | pyrazinamide.ti,ab. (1,970) | S30 | (TI ethambutol) OR (AB ethambutol) [307] |
| 31 | rimactane.ti,ab. (16) | 31 | tubazide.ti,ab. (15) | 31 | or/13-30 (16,293) | S31 | (TI myambutol) OR (AB myambutol) [2] |
| 32 | Ethambutol/ (3,721) | 32 | rifampin.ti,ab. (9,266) | 32 | 6 and 12 and 31 (627) | S32 | (TI pyrazinamide) OR (AB pyrazinamide) [280] |
| 33 | ethambutol.ti,ab. (4,686) | 33 | benemycin.ti,ab. (19) | 33 | limit 32 to yr=”1990 -Current” (452) | S33 | S15 OR S16 OR S17 OR S18 OR S19 OR S20 OR S21 OR S22 OR S23 OR S24 OR S25 OR S26 OR S27 OR S28 OR S29 OR S30 OR S31 OR S32 [2,859] |
| 34 | myambutol.ti,ab. (42) | 34 | rifadin.ti,ab. (77) | 34 | remove duplicates from 33 (452) | S34 | S8 AND S14 AND S33 [147] |
| 35 | Pyrazinamide/ (3,028) | 35 | rifampicin.ti,ab. (18,669) |  |  |  |  |
| 36 | pyrazinamide.ti,ab. (3,413) | 36 | rimactan.ti,ab. (46) |  |  |  |  |
| 37 | or/19-36 (45,334) | 37 | rimactane.ti,ab. (19) |  |  |  |  |
| 38 | 8 and 18 and 37 (1,339)) | 38 | ethambutol.ti,ab. (6,693) |  |  |  |  |
| 39 | limit 38 to yr= "1990 -Current" (814) | 39 | myambutol.ti,ab. (62) |  |  |  |  |
| 40 | remove duplicates from 39 (808) | 40 | pyrazinamide.ti,ab. (4,562) |  |  |  |  |
|  |  | 41 | or/16-40 (114,515) |  |  |  |  |
|  |  | 42 | 8 and 15 and 41 (1,845) |  |  |  |  |
|  |  | 43 | limit 42 to yr=”1990 -Current” (1,510) |  |  |  |  |
|  |  | 44 | remove duplicates from 43 (1,463) |  |  |  |  |
