# Supplementary material for: Dynamics of sputum conversion during effective tuberculosis treatment: A systematic review and meta-analysis
Source: PLoS Med. 2021 Apr 26;18(4):e1003566. doi: 10.1371/journal.pmed.1003566 (PMC8109831; doi:10.1371/journal.pmed.1003566)
Supplement: S3 Table — (DOCX) [file pmed.1003566.s008.docx]

| S3 Table. Details of databases searched and terms used for animal studies search; run on March 27^th^ 2018 |
| --- |
| NCBI Pubmed  Date: full database searched |
| (infectiousness [All Fields] OR (“transmission”[Subheading] OR “transmission”[All Fields])) AND ((“tuberculosis”[MeSH Terms] OR “tuberculosis”[All Fields]) AND (“therapy”[Subheading] OR “therapy”[All Fields] OR “treatment”[All Fields] OR “therapeutics”[MeSH Terms] OR “therapeutics”[All Fields])) AND “animals”[MeSH Terms: noexp] |
