# Supplementary material for: Dynamics of sputum conversion during effective tuberculosis treatment: A systematic review and meta-analysis
Source: PLoS Med. 2021 Apr 26;18(4):e1003566. doi: 10.1371/journal.pmed.1003566 (PMC8109831; doi:10.1371/journal.pmed.1003566)
Supplement: S4 Table — (DOCX) [file pmed.1003566.s009.docx]

| S4 Table. Summary of quality scores for included primary research studies, assessed using an adapted NIH tool for case series (N = 44 studies) [1]. | | | | | | | | | | | |
| --- | --- | --- | --- | --- | --- | --- | --- | --- | --- | --- | --- |
| First author, year published | Was the study question or objective clearly stated? | Was the study population clearly and fully described, including a case definition? | Were the cases consecutive or randomly allocated in an RCT? | Were the subjects comparable? | Was the intervention clearly described? | Were the outcome measures clearly defined, valid, reliable, and implemented consistently? | Was the length of follow-up adequate (at least 2 months, 90% retention)? | Were the statistical methods well-described? | Were the results well-described? | Total (/9) | Quality Rating |
| Abal, 2005 | 1 | 0 | 1 | 0 | 1 | 1 | 1 | 1 | 1 | **7** | **Good** |
| BTA, 1981 | 1 | 1 | 1 | 1 | 1 | 1 | 1 | 1 | 1 | **9** | **Good** |
| Chaulet, 1995 | 1 | 1 | 1 | 1 | 1 | 1 | 1 | 1 | 1 | **9** | **Good** |
| Combs, 1990 | 1 | 1 | 1 | 1 | 1 | 1 | 1 | 1 | 0 | **8** | **Good** |
| Conde, 2009 | 1 | 1 | 1 | 1 | 1 | 1 | 1 | 1 | 1 | **9** | **Good** |
| Conde, 2016 | 1 | 1 | 1 | 1 | 1 | 1 | 1 | 1 | 1 | **9** | **Good** |
| Dawson, 2009 | 1 | 1 | 1 | 1 | 0 | 1 | 1 | 1 | 1 | **8** | **Good** |
| Dawson, 2015a | 1 | 1 | 1 | 1 | 0 | 1 | 1 | 1 | 1 | **8** | **Good** |
| Dawson, 2015b | 1 | 1 | 1 | 1 | 1 | 1 | 1 | 1 | 0 | **8** | **Good** |
| DesJardin, 1999 | 1 | 1 | 0 | 0 | 0 | 1 | 0 | 1 | 1 | **5** | **Fair** |
| Dlugovitzky, 2006 | 0 | 0 | 0 | 0 | 1 | 1 | 1 | 0 | 1 | **4** | **Fair** |
| Dominguez-Castellano, 2003 | 1 | 1 | 1 | 1 | 0 | 1 | 1 | 1 | 1 | **8** | **Good** |
| Dorman, 2009 | 1 | 1 | 0 | 1 | 1 | 1 | 1 | 1 | 1 | **8** | **Good** |
| Dorman, 2012 | 1 | 1 | 1 | 1 | 1 | 1 | 0 | 1 | 1 | **8** | **Good** |
| Dorman, 2015 | 1 | 1 | 1 | 1 | 1 | 1 | 1 | 1 | 1 | **9** | **Good** |
| E&C Africa / BMRC, 1983 | 1 | 1 | 1 | 1 | 1 | 1 | 1 | 0 | 1 | **8** | **Good** |
| Grandjean 2015 | 1 | 1 | 1 | 1 | 1 | 1 | 1 | 1 | 1 | **9** | **Good** |
| HKCS / BMRC, 1978 | 1 | 1 | 1 | 1 | 1 | 1 | 1 | 1 | 1 | **9** | **Good** |
| HKCS / BMRC, 1981 | 1 | 0 | 1 | 1 | 1 | 1 | 1 | 0 | 1 | **7** | **Good** |
| Jindani, 2014 | 1 | 1 | 1 | 1 | 1 | 1 | 1 | 1 | 1 | **9** | **Good** |
| Jindani, 2016 | 1 | 1 | 1 | 1 | 1 | 1 | 1 | 1 | 1 | **9** | **Good** |
| Johnson, 2000 | 1 | 1 | 1 | 1 | 1 | 1 | 1 | 1 | 1 | **9** | **Good** |
| Johnson, 2003 | 1 | 1 | 1 | 1 | 1 | 1 | 1 | 1 | 1 | **9** | **Good** |
| Joloba, 2000 | 1 | 1 | 0 | 1 | 1 | 1 | 1 | 1 | 1 | **8** | **Good** |
| Kanda, 2015 | 1 | 1 | 1 | 0 | 1 | 1 | 1 | 1 | 1 | **8** | **Good** |
| Kennedy, 1996 | 1 | 1 | 1 | 1 | 1 | 1 | 1 | 1 | 1 | **9** | **Good** |
| Lee, 2014 | 1 | 1 | 1 | 1 | 0 | 1 | 0 | 1 | 1 | **7** | **Good** |
| Leung, 2017 | 1 | 1 | 1 | 1 | 0 | 1 | 1 | 1 | 1 | **8** | **Good** |
| Long, 2003 | 1 | 1 | 1 | 1 | 0 | 1 | 0 | 1 | 0 | **6** | **Fair** |
| Mechai, 2016 | 1 | 1 | 0 | 1 | 1 | 1 | 1 | 1 | 1 | **8** | **Good** |
| Musteikiene, 2017 | 1 | 1 | 1 | 1 | 1 | 1 | 1 | 1 | 1 | **9** | **Good** |
| Pheiffer, 2008 | 1 | 0 | 0 | 0 | 0 | 1 | 0 | 1 | 1 | **4** | **Fair** |
| Rathored, 2012 | 1 | 0 | 1 | 0 | 0 | 0 | 0 | 1 | 1 | **4** | **Fair** |
| Sajid, 2011 | 1 | 1 | 1 | 0 | 1 | 1 | 1 | 1 | 1 | **8** | **Good** |
| Scott, 2017 | 1 | 1 | 1 | 1 | 1 | 1 | 1 | 1 | 1 | **9** | **Good** |
| STBS / BMRC, 1979 | 1 | 0 | 1 | 1 | 1 | 1 | 1 | 0 | 1 | **7** | **Good** |
| STBS / BMRC, 1985 | 1 | 1 | 1 | 1 | 1 | 1 | 1 | 1 | 1 | **9** | **Good** |
| Singla, 2003 | 1 | 1 | 1 | 0 | 1 | 1 | 1 | 1 | 1 | **8** | **Good** |
| Stoffel, 2014 | 1 | 0 | 1 | 1 | 1 | 1 | 1 | 1 | 0 | **7** | **Good** |
| Tanzania / BMRC, 1985 | 1 | 1 | 1 | 1 | 1 | 1 | 1 | 1 | 1 | **9** | **Good** |
| Tanzania / BMRC, 1996 | 1 | 1 | 1 | 1 | 1 | 1 | 0 | 0 | 0 | **6** | **Fair** |
| TBRC, 1983 | 0 | 1 | 1 | 1 | 1 | 1 | 1 | 0 | 1 | **7** | **Good** |
| Telzak, 1997 | 1 | 0 | 1 | 1 | 0 | 1 | 1 | 1 | 1 | **7** | **Good** |
| Volkmann, 2015 | 1 | 1 | 1 | 1 | 0 | 0 | 0 | 1 | 1 | **6** | **Fair** |
| **Abbreviations:**  1= yes;  0 = no;  BMRC = British Medical Research Council;  BTA = British Thoracic Association;  HKCS = Hong Kong Chest Service;  STBS = Singapore Tuberculosis Service;  TBRC = Tuberculosis Research Centre. | | | | | | | | | | | |
|  | | | | | | | | | | | |

1. National Institutes of Health. Study Quality Assessment Tools: Quality Assessment Tool for Case Series Studies. Available from: https://www.nhlbi.nih.gov/health-topics/study-quality-assessment-tools. Accessed 9 January 2020.
