# Supplementary material for: Dynamics of sputum conversion during effective tuberculosis treatment: A systematic review and meta-analysis
Source: PLoS Med. 2021 Apr 26;18(4):e1003566. doi: 10.1371/journal.pmed.1003566 (PMC8109831; doi:10.1371/journal.pmed.1003566)
Supplement: S5 Table — (DOCX) [file pmed.1003566.s010.docx]

| S5 Table. Studies reporting a summary measure of time to culture conversion where the culture type was not specified* | | | | | | | |
| --- | --- | --- | --- | --- | --- | --- | --- |
| **First author** | **Year** | **Country** | **Smear+** | **Sampling frequency** | **N** | **Median, days**  **(IQR)** | **Mean, days**  **(range/SE)** |
| Kennedy | 1996 | Tanzania | 100% | monthly | 81 | - | 55 (range 30–120) |
| Telzak | 1997 | USA | 100% | weekly | 65 | - | 32 ± 14 (2 SE) |
| Volkmann | 2015 | USA | NS  NS | NS  NS | 46,925†  12,512‡ | 43 (25-66)  54 (31-76) | -  - |
| Mechai | 2016 | France | 100% | fortnightly^\|\|^ | 18 | 48 (26-58) | - |
| **Abbreviations:**  N = number assessed for outcome  NS = not stated;  IQR = interquartile range;  SE = standard error;  USA = United States of America;  - = data not available  **Footnotes:**  * Where authors reported data in months these have been converted to days to aid comparisons using 30.4 days per month.  † patients reporting no excess alcohol use;  ‡ patients reporting excess alcohol use;  ^\|\|^ on days 7,8 and 9 then on 3 consecutive days every two weeks. | | | | | | | |
