# Supplementary material for: Dynamics of sputum conversion during effective tuberculosis treatment: A systematic review and meta-analysis
Source: PLoS Med. 2021 Apr 26;18(4):e1003566. doi: 10.1371/journal.pmed.1003566 (PMC8109831; doi:10.1371/journal.pmed.1003566)
Supplement: Attachment — Submitted filename: PMEDICINE-D-20-03202_R1_reviewer.pdf [file pmed.1003566.s011.pdf]

PLOS Medicine

Sputum conversion during tuberculosis treatment: systematic review and meta-analysis

--Manuscript Draft--

**Manuscript Number:**	PMEDICINE-D-20-03202R1
**Full Title:**	Sputum conversion during tuberculosis treatment: systematic review and meta-analysis
**Short Title:**	Sputum conversion during tuberculosis treatment: systematic review and meta-analysis
**Article Type:**	Research Article
**Abstract:**	Rationale: Two weeks' isolation is widely recommended for people commencing treatment for pulmonary tuberculosis. The evidence that this corresponds to clearance of potentially infectious tuberculous mycobacteria in sputum is not well established.<br>Objectives: This World Health Organization-commissioned review investigated sputum sterilization dynamics during tuberculosis treatment.<br>Methods: Systematic literature searches were conducted before duplicate sifting and data extraction. For the main analysis, included articles reported on patients receiving effective treatment for drug-sensitive pulmonary tuberculosis. The outcome of interest was sputum bacteriological conversion: the proportion of patients having converted by a defined timepoint or a summary measure of time to conversion. Random effects meta-analyses were performed. A narrative summary additionally describes data evaluating infectiousness of humans to experimental animals.<br>Results: Of 5,290 identified records, 44 were included. Summary estimates suggested smear conversion in 9% of patients at two weeks and 82% at two months of treatment (95% confidence interval (CI), 3–24% and 78–86%, study numbers (N) = 1 and 10). Among baseline smear-positive patients, solid culture conversion occurred by two weeks in 5% (CI 0–14%, N=2), increasing to 88% (CI 84–92%, N=20) at two months. At equivalent timepoints, liquid culture conversion was achieved in 3% (CI 1–16%, N=1) and 59% (CI 47–70%, N=8). Significant heterogeneity was observed.<br>Conclusions: Most patients remained culture-positive at two weeks, challenging the view that individuals are not infectious after this interval. Culture-positivity is, however, only one component of infectiousness: other factors such as cough dynamics are important.
**Additional Information:**	
**Question**	**Response**
**Financial Disclosure**<br><br>Enter a financial disclosure statement that describes the sources of funding for the work included in this submission. Review the submission guidelines for detailed requirements. View published research articles from *PLOS Medicine* for specific examples.<br><br>This statement is required for submission and **will appear in the published article** if the submission is accepted. Please make sure it is accurate.	This work was partly supported by a contract to the LSHTM TB Centre from the Global TB Programme of the World Health Organization<br><br>Authors received a small compensation for their time, with funds allocated from the WHO contract.<br><br>No formal grants were awarded.

**Unfunded studies**<br>Enter: *The author(s) received no specific funding for this work.*<br><br>**Funded studies**<br>Enter a statement with the following details:<br>• Initials of the authors who received each award<br>• Grant numbers awarded to each author<br>• The full name of each funder<br>• URL of each funder website<br>• Did the sponsors or funders play any role in the study design, data collection and analysis, decision to publish, or preparation of the manuscript?<br>• **NO** - Include this sentence at the end of your statement: *The funders had no role in study design, data collection and analysis, decision to publish, or preparation of the manuscript.*<br>• **YES** - Specify the role(s) played.<br><br>* typeset	
**Competing Interests**<br><br>Use the instructions below to enter a competing interest statement for this submission. On behalf of all authors, disclose any competing interests that could be perceived to bias this work—acknowledging all financial support and any other relevant financial or non-financial competing interests.<br><br>This statement **will appear in the published article** if the submission is accepted. Please make sure it is accurate. View published research articles from *PLOS Medicine* for specific examples.	The authors have declared that no competing interests exist

**NO authors have competing interests**<br><br>Enter: *The authors have declared that no competing interests exist.*<br><br>**Authors with competing interests**<br>Enter competing interest details beginning with this statement:<br><br>*I have read the journal's policy and the authors of this manuscript have the following competing interests: [insert competing interests here]*<br><br>* typeset	
**Ethics Statement**<br><br>You must include an Ethics Statement in the Methods section of your manuscript if your study involved:<br>• Human participants<br>• Human specimens or tissue<br>• Vertebrate animals or cephalopods<br>• Vertebrate embryos or tissues<br>• Field research<br><br>**Not including a statement when required will delay the review process.**<br><br>General guidance is provided below. Please consult the guidelines on human subjects research and animal research for detailed instructions.	I confirm that I have read and understood PLOS' requirements for an Ethics Statement.

Powered by Editorial Manager® and ProduXion Manager® from Aries Systems Corporation

**Format for specific study types**<br><br>**Human Subject Research (involving human participants and/or tissue)**<br>• Give the name of the institutional review board or ethics committee that approved the study<br>• Include the approval number and/or a statement indicating approval of this research<br>• Indicate the form of consent obtained (written/oral) or the reason that consent was not obtained (e.g. the data were analyzed anonymously)<br><br>**Animal Research (involving vertebrate animals, embryos or tissues)**<br>• Provide the name of the Institutional Animal Care and Use Committee (IACUC) or other relevant ethics board that reviewed the study protocol, and indicate whether they approved this research or granted a formal waiver of ethical approval<br>• Include an approval number if one was obtained<br>• If the study involved *non-human primates*, add *additional details* about animal welfare and steps taken to ameliorate suffering<br><br>**Field Research**<br><br>Include the following details if this study involves the collection of plant, animal, or other materials from a natural setting:<br>• Field permit number<br>• Name of the institution or relevant body that granted permission<br><br>Please check the box to confirm your understanding of this policy, then read and agree to one of the statements.	
Please respond:<br>as follow-up to "**Ethics Statement**<br><br>You must include an Ethics Statement in the Methods section of your manuscript if your study involved:<br>• Human participants	My study does not require an ethics statement.

• Human specimens or tissue<br>• Vertebrate animals or cephalopods<br>• Vertebrate embryos or tissues<br>• Field research<br><br>**Not including a statement when required will delay the review process.**<br><br>General guidance is provided below. Please consult the guidelines on human subjects research and animal research for detailed instructions.	

Powered by Editorial Manager® and ProduXion Manager® from Aries Systems Corporation

**Format for specific study types**<br><br>**Human Subject Research (involving human participants and/or tissue)**<br>• Give the name of the institutional review board or ethics committee that approved the study<br>• Include the approval number and/or a statement indicating approval of this research<br>• Indicate the form of consent obtained (written/oral) or the reason that consent was not obtained (e.g. the data were analyzed anonymously)<br><br>**Animal Research (involving vertebrate animals, embryos or tissues)**<br>• Provide the name of the Institutional Animal Care and Use Committee (IACUC) or other relevant ethics board that reviewed the study protocol, and indicate whether they approved this research or granted a formal waiver of ethical approval<br>• Include an approval number if one was obtained<br>• If the study involved *non-human primates*, add *additional details* about animal welfare and steps taken to ameliorate suffering<br><br>**Field Research**<br><br>Include the following details if this study involves the collection of plant, animal, or other materials from a natural setting:<br>• Field permit number<br>• Name of the institution or relevant body that granted permission<br><br>Please check the box to confirm your understanding of this policy, then read and agree to one of the statements. "	
**Data Availability**<br><br>Authors are required to make all data underlying the findings described fully available, without restriction, and from the time of publication. PLOS allows rare exceptions to address legal and ethical concerns. See the PLOS Data Policy and	Yes - all data are fully available without restriction

Powered by Editorial Manager® and ProduXion Manager® from Aries Systems Corporation

FAQ for detailed information.<br><br>A Data Availability Statement describing where the data can be found is required at submission. Your answers to this question constitute the Data Availability Statement and **will be published in the article**, if accepted.<br><br>**Important:** Stating 'data available on request from the author' is not sufficient. If your data are only available upon request, select 'No' for the first question and explain your exceptional situation in the text box.<br><br>Do the authors confirm that all data underlying the findings described in their manuscript are fully available without restriction?	
**Describe where the data may be found in full sentences. If you are copying our sample text, replace any instances of XXX with the appropriate details.**<br><br>• If the data are **held or will be held in a public repository**, include URLs, accession numbers or DOIs. If this information will only be available after acceptance, indicate this by ticking the box below. For example: *All XXX files are available from the XXX database (accession number(s) XXX, XXX.*).<br>• If the data are all contained **within the manuscript and/or Supporting Information files**, enter the following: *All relevant data are within the manuscript and its Supporting Information files.*<br>• If neither of these applies but you are able to provide **details of access elsewhere**, with or without limitations, please do so. For example:<br><br>*Data cannot be shared publicly because of [XXX]. Data are available from the XXX Institutional Data Access / Ethics Committee (contact via XXX) for*	All relevant data are within the manuscript and its Supporting Information files.

researchers who meet the criteria for access to confidential data.<br><br>*The data underlying the results presented in the study are available from (include the name of the third party and contact information or URL).*<br>• This text is appropriate if the data are owned by a third party and authors do not have permission to share the data.<br><br>* typeset	
Additional data availability information:	Tick here if your circumstances are not covered by the questions above and you need the journal’s help to make your data available.

Powered by Editorial Manager® and ProduXion Manager® from Aries Systems Corporation

Manuscript
Click here to access/download;Manuscript;manuscript_PLOSM_submission

Sputum conversion during tuberculosis treatment: systematic review and meta-analysis

Authors
Claire J Calderwood*[1]
James P Wilson*[1]
Katherine L Fielding[1]
Rebecca C Harris[1]
Aaron S Karat[1]
Raoul Mansukhani[1]
Jane Falconer[2]
Malin Bergström[1]
Sarah M Johnson[1]
Nicky McCreesh[1]
Edward JM Monk[1]
Jasantha Odayar[3]
Peter J Scott[1]
Sarah A Stokes[1]
Hannah Theodorou[1]
David AJ Moore[1]
* these authors contributed equally to the manuscript

Affiliations
1) TB Centre, London School of Hygiene & Tropical Medicine, Keppel St, London WC1E 7HT, UK
2) Library & Archives Service, London School of Hygiene and Tropical Medicine, Keppel St, London WC1E 7HT, UK
3) Division of Epidemiology and Biostatistics, School of Public Health & Family Medicine, University of Cape Town, Cape Town, South Africa 7925

Corresponding author
David AJ Moore. TB Centre, London School of Hygiene & Tropical Medicine, Keppel St, London WC1E 7HT, UK

Author contributions
CJ Calderwood, JP Wilson: Literature search, data extraction, analysis and interpretation. Contributions to study conception, design and manuscript drafting.
KL Fielding: Support in overall study conception and design, statistical oversight.
RC Harris, AS Karat: Data extraction, analysis and interpretation
R Mansukhani: Data analysis
J Falconer: Literature searching
M Bergström, SM Johnson, N McCreesh, EJM Monk, J Odayar, P Scott, SA Stokes, H Theodorou: Data extraction
DAJ Moore: Overall oversight of study, design, data extraction and interpretation and manuscript drafting.

Support
This work was partly supported by a contract to the LSHTM TB Centre from the Global TB Programme of the World Health Organization

Total word count: 3082

Abstract

Rationale: Two weeks' isolation is widely recommended for people commencing treatment for pulmonary tuberculosis. The evidence that this corresponds to clearance of potentially infectious tuberculous mycobacteria in sputum is not well established.

Objectives: This World Health Organization-commissioned review investigated sputum sterilization dynamics during tuberculosis treatment.

Methods: Systematic literature searches were conducted before duplicate sifting and data extraction. For the main analysis, included articles reported on patients receiving effective treatment for drug-sensitive pulmonary tuberculosis. The outcome of interest was sputum bacteriological conversion: the proportion of patients having converted by a defined timepoint or a summary measure of time to conversion. Random effects meta-analyses were performed. A narrative summary additionally describes data evaluating infectiousness of humans to experimental animals.

Results: Of 5,290 identified records, 44 were included. Summary estimates suggested smear conversion in 9% of patients at two weeks and 82% at two months of treatment (95% confidence interval (CI), 3–24% and 78–86%, study numbers (N) = 1 and 10). Among baseline smear-positive patients, solid culture conversion occurred by two weeks in 5% (CI 0–14%, N=2), increasing to 88% (CI 84–92%, N=20) at two months. At equivalent timepoints, liquid culture conversion was achieved in 3% (CI 1–16%, N=1) and 59% (CI 47–70%, N=8). Significant heterogeneity was observed.

Conclusions: Most patients remained culture-positive at two weeks, challenging the view that individuals are not infectious after this interval. Culture-positivity is, however, only one component of infectiousness: other factors such as cough dynamics are important.

Abstract word count: 249

Key words: transmission, prevention, infection control, microscopy, culture

Introduction

Tuberculosis (TB) is the leading cause of death from an infectious disease.(1) *Mycobacterium tuberculosis* (*Mtb*), the causative organism, is transmitted via respiratory droplet nuclei generated by individuals with pulmonary TB disease (PTB). Transmission is affected by environmental factors, characteristics of both the source case and exposed individual, and the nature of their contact. The greatest risk is from individuals with smear-positive PTB and high cough frequency.(2–4) Interruption of transmission requires identification and separation of infectious individuals until they are rendered non-infectious through treatment. National guidelines recommend isolation of hospitalised individuals, with two weeks of effective treatment commonly cited as the timeframe after which patients are considered non-infectious.(5–8) Implementing respiratory isolation has considerable resource implications, and is often unachievable in overstretched health systems in low and middle-income countries where the greatest burden of TB lies.(1)

The evidence-base for the two-week 'rule' has been repeatedly challenged by data demonstrating that most baseline sputum culture-positive patients positive remain so for longer than 14 days.(9) These individuals are potentially infectious, although diminished cough frequency may reduce transmission.(4, 10)

This systematic review was commissioned by the World Health Organization (WHO) Global TB Department Guideline Development Group (GDG) to synthesise evidence on the dynamics of potential infectiousness of individuals with PTB receiving effective therapy; informing the 2019 guideline update on TB infection prevention and control (IPC).(11)

Potential infectiousness was defined here as detection of *Mtb* by sputum smear or culture, irrespective of cough dynamics or contact mixing patterns. Whilst smear microscopy may detect non-viable, and therefore non-infectious bacilli, it is referenced as a measure of treatment response in published guidelines and in clinical practice. Molecular methods (*Mtb* DNA detection, e.g. Xpert MTB/RIF®) also detect non-viable bacilli and are not routinely used in assessment of treatment response; such assessments were excluded from this analysis.

Two types of evidence were considered: (1) data on time from treatment initiation to sputum smear and culture conversion, and (2) data from measurements of infectiousness of PTB patients to experimental animals. Although cough is clearly important, review of cough dynamics during TB treatment was outside the scope of this study.

Methods

Search strategy

This review was conducted following Preferred Reporting Items for Systematic Reviews and Meta-Analyses (PRISMA) guidelines(12). A professional librarian developed and executed the literature search, in consultation with the WHO GDG. The population of interest was patients receiving an effective treatment regimen for drug-susceptible PTB (DS-TB); the outcome of interest was bacteriological conversion of sputum. Effective treatment required at least rifampicin (R) and isoniazid (H) throughout, pyrazinamide (Z) for the 2-month intensive phase, and administration at least 5 days per week. Inclusion of ethambutol (E) and streptomycin (S) was permitted but not essential. Regimens including drugs not conventionally regarded as first-line, such as quinolones, were excluded.(13) The outcome

was defined as either the proportion of participants achieving bacteriological (smear or culture) conversion from positive to negative by fixed timepoints during treatment (proportion converted; PC) or time to conversion (TTC) for the study population. As a descriptive analysis, no intervention or comparator group was defined.

Full search parameters are detailed in the online supplement (Tables E1-2). The search was constructed in OvidSP MEDLINE, adapted and run in OvidSP EMBASE, OvidSP Global Health and EBSCO CINAHL Plus. Searches used subject headings where available, and search terms run in the title and abstract. Combinations of terms were used to capture the concepts of infectiousness and tuberculosis. Language was limited to English, Japanese, Chinese, Russian, French, Spanish and Portuguese. On initial screening of search results, the authors noted the absence of several randomised controlled trials (RCTs) known to include culture conversion data in standard treatment arms. An additional search was therefore constructed identifying all RCTs of DS-TB treatment in humans. The first search was conducted on 1st December 2017 without date restrictions; the second (RCT search) was conducted on 20th February 2018 and date limited to 1990 onwards to identify RCTs incorporating standard daily rifampicin-based regimens.

Study selection and data extraction

Two-stage sifting by two independent reviewers was employed, applying pre-determined eligibility criteria at title, abstract, and where necessary, full text screening. References and citations for all included studies were reviewed to identify further relevant articles. Table 1 details comprehensive inclusion and exclusion criteria.

[Table 1]

Data were extracted in duplicate into a standardised, pilot-tested Microsoft Excel® database. As our intention was to analyse an 'on treatment' population, data from per-protocol analyses were preferentially used. Data were not directly extracted from systematic reviews; instead, source manuscripts were identified and reviewed for inclusion. At each stage of screening and extraction, disagreements were resolved by a third independent reviewer [DAJM].

Quality assessment was performed by two independent reviewers at study level using an adapted National Institutes of Health tool for case series.(14)

Analysis

Extracted data were synthesised using a narrative approach, and meta-analyses where appropriate. TTC data were presented as study-specific estimates (mean or median) together with measure of spread (interquartile range, standard error, standard deviation or range) where provided.

Random-effect meta-analyses were used to calculate pooled conversion proportions across detection method subgroups (smear, solid culture or liquid culture) at different time-points. Where the culture type was not stated, those performed prior to 1990 were assumed to use solid media, others are reported as 'unspecified'. Study-specific CIs were calculated using the score method. Weighted pooled proportion estimates were calculated for each subgroup using the Freeman-Tukey double arcsine transformation, avoiding potential bias introduced from excluding studies with conversion proportions equalling 0 or 1.(15) Pooled estimate CIs

were calculated using the Wald method. Within-subgroup heterogeneity was assessed with the chi-squared test and described with the I^2 statistic. Data cleaning was conducted in Stata (Stata/IC version 13.1, Stata Corp., College Station, TX, USA) and meta-analyses performed in R (R Core Team, 2020) using the 'forest.meta' function from the package 'meta'.(16)

Animal studies

A review of data on *Mtb* transmission from humans to animals was explicitly requested by the WHO GDG and undertaken as a separate search and analysis. The population of interest was patients receiving effective treatment for drug-susceptible or drug-resistant PTB. The outcome of interest was transmission to experimental animals exposed to air exhausted from isolation facilities accommodating TB patients, defined as tuberculin skin test (TST) conversion or new active TB disease in these animals.

Search details are described in the online supplement (Table E3). Titles and abstracts, then full texts of identified papers were reviewed. References and citations of included papers were also reviewed. The diversity and heterogeneity of methodologies and analyses applied by included studies did not permit standardised data extraction or quality assessment. Relevant data were extracted for narrative synthesis by one reviewer and independently checked for accuracy by a second.

Results

In total, 5,290 unique records were identified for title and abstract review (first search: 3,558 and RCT search: 1,732). Full text review of 180 papers identified 22 studies for inclusion in

the main analysis. A further 22 articles were added from references and citations (Figure 1) resulting in 44 papers for analysis (Table 2).

[Figure 1]

Of the included papers, 27 were RCTs and 17 were cohort studies (Table 1). Thirteen studies (30%) reported data from Africa, 12 from Asia (27%), ~~6~~ from South America (14%), ~~5~~ from North America (11%) and ~~4~~ from Europe (9%). Four studies reported data from multiple continents. Intensive phase treatment in most studies was comprised of RHZE (31 studies); ~~6~~ studies used streptomycin instead of ethambutol, the remainder used other acceptable regimens (RHZ with or without E or S). Seventeen studies included people living with HIV, representing between 0.8 and 61% of the study population. Thirty-three studies only included people with baseline ~~smear positive~~ PTB.

[Table 2]

Eight studies reported both PC and TTC data; 31 studies reported only PC data and ~~5~~ reported only TTC data. For data on the proportion of patients achieving bacteriological conversion, there were a total of 96 estimates; ~~4, 5 and 6~~ estimates at 1, 2 and 3 weeks of treatment respectively; and 20, 46, 7 and 8 estimates at 1, 2, 3 and 4 months respectively (Table 3).

[Table 3]

~~37~~ studies were ranked as “good” in the quality scoring system (>7/9), with ~~7~~ studies ranked as “fair” (4-6/9), and none as “poor” quality (supplementary material, Table E7).

Smear conversion

Fifteen studies reporting data on smear conversion were included, providing 18 estimates of the proportion of individuals who had smear converted at defined time points on treatment and 6 summary estimates of the time to smear conversion.

For the 6 studies reporting a summary estimate of time to smear conversion the median time to conversion ranged from 20 to 27 days and the mean time to conversion ranged from 29 to 55 days (Table 4). Shorter times to conversion were seen in studies with more frequent sampling.

[Table 4]

The proportion of treated patients with baseline smear-positive DS-TB achieving positive-to-negative smear conversion at months 1, 2, 3 and 4 are shown in Figure 2.

From a total of 18 estimates across 11 studies, the proportion of baseline smear-positive patients achieving smear conversion at the 1-, 2-, 3- and 4-month time points were 33% (95% CI 25–42%), 82% (95% CI 78–86%), 94% (95% CI 94-95%) and 100% (95%CI 72-100%) respectively.

[Figure 2]

Culture conversion

Thirty-six studies reported data on culture conversion; ~~9~~ using liquid culture and 23 using solid (~~8~~ studies reported both). For 12 studies the method of culture was unspecified and these data are presented in the online supplement (Table E4 and Figure E1).

Summary statistics for TTC were reported in 11 studies and are summarised by culture medium in Table 5. All studies reporting solid TTC data (N=6) included only smear-positive patients at baseline. The median TTC ranged from 35 to 49 days and the mean TTC ranged from 24 to 46 days.

Four studies reported a summary estimate of liquid TTC. Median TTC ranged from 40 to 59 days among baseline smear-positive patients. One study stratified liquid culture TTC by smear status, with a median TTC of 19 days in baseline smear-negative patients compared to 40 days for smear-positive patients.

[Table 5]

The proportion of treated patients with DS-TB achieving positive-to-negative culture conversion at weeks 1, 2 and 3, and months 1, 2, 3, and 4 (77 estimates in total) are shown in Figures 3 and 4 for solid and liquid culture methods, respectively.

[Figure 3]

[Figure 4]

Most PC data are available at the 2-month time point, where treatment steps down from intensive to maintenance phase. Figure 5 illustrates the proportion of patients achieving culture conversion at 2 weeks and 2 months, restricted to only ~~including~~ patients with baseline smear-positive samples.

[Figure 5]

The proportion of patients treated for DS-TB achieving smear and culture conversion at each time point is summarised in Table 6.

[Table 6]

Animal studies

The search to identify relevant animal studies returned 646 results from which ~~3~~ were found to be relevant at title and abstract sifting; all met inclusion criteria at full-text sifting. A fourth study (Riley et al, 1962)(17) was added from reference and citation checking of the included articles (Figure 6, Table 7).

[Table 7]

These four papers all describe experimental data from studies in which groups of guinea pigs, used as air samplers, were exposed to air exhausted from dedicated isolation rooms accommodating PTB patients. Three studies evaluated the infectiousness of patients with DS-TB(17–19) to experimental animals and one considered patients with multi-drug resistant TB.(20) None of the experiments were designed to evaluate temporal changes in infectiousness during TB treatment, however Dharmadhikari et al. re-analysed five previous

experiments with this objective.(20) As such the available data are opportunistically available and incomplete, and it is not possible to determine the magnitude of incompleteness. Due to considerable methodological and analytical differences between studies, aggregation of findings was not possible. A brief narrative summary of each individual paper is provided in the online supplement.

Overall, the three papers evaluating infectiousness of air from patients with DS-TB suggested that those on treatment were less infectious to guinea pigs than untreated patients, although a small number of infections did occur during effective treatment. There are no data that describe how infectiousness changed over time after treatment initation. It was not possible to disaggregate guinea pig infections occuring from patients with drug-resistant TB on or off effective treatment in the paper by Dharmadhikari et al. It is not possible from these data to draw conclusions about the role of effective treatment in reducing infectiveness of TB, nor on the time-frame over which any reduction in infectiousness occurs.

Discussion

Identification of people with active tuberculosis and initiation of effective treatment is a vital component of infection control, mediated through what is conventionally perceived as a rapid reduction in the infectiousness of the treated patient. As part of the evidence gathering for the 2019 WHO Guidelines on TB IPC,(11) we undertook a systematic review and meta-analysis to investigate the dynamics of sputum sterilisation after initiation of effective treatment.

This review draws together the best currently available data on the dynamics of sputum sterilisation during effective TB treatment, considering several assessment methods: sputum smear, and both solid and liquid culture conversion. The evaluation of TB transmission from human patients to experimental animals provides an additional perspective.

We show that despite an optimal drug regimen for DS-TB, approximately 1 in 5 baseline smear-positive patients do not achieve smear conversion by 2 months of treatment; decreasing to 1 in 20 by 3 months. It is important for clinicians and programmes to appreciate this significant tail of individuals who, despite receiving effective therapy, remain smear-positive.

After 2 weeks of effective treatment, 95% and 97% of baseline smear-positive patients demonstrate persistently positive solid and liquid cultures respectively. This directly challenges guidance that infection, prevention and control measures can be relaxed following 2 weeks of effective treatment. On completion of the 2-month intensive treatment phase, 12% and 41% of baseline smear-positive patients remain solid and liquid culture-positive respectively.

Assessment of the capacity to detect *Mtb* in spontaneously produced sputum does not directly quantify infectiousness, which also depends on environment, cough dynamics and recipient factors. The core of this analysis relates to culture conversion as this is an assessment of presence of viable *Mtb* in respiratory samples; we included data on smear conversion as the most frequently used microbiological test used for response to therapy, however smear-positivity of treated patients may not reflect presence of viable *Mtb*.

Presence of *Mtb* in respiratory secretions is necessary, although in the absence of a mechanism for propagation, not sufficient for infectiousness. Cough frequency diminishes significantly in the first few weeks of effective TB treatment; this likely plays an important role in reducing transmission risk.(4, 10)

The presented estimates should be interpreted with consideration of the heterogeneity within and between studies. TB patients vary widely in terms of gender, age, exposure history, disease duration and severity (including smear status and presence or absence of lung cavitation), *Mtb* strain lineage, nutritional status, profile of comorbidities such as HIV and diabetes, and tolerance of and adherence to treatment. Aggregation of TB patients into study populations magnifies rather than diminishes this heterogeneity; there is no "typical" cohort of TB patients. Many of these covariates are independently associated with time to sputum conversion(21, 22): when inferring a relationship between detection method and conversion estimates this often nuanced confounding should be recognised.

In our analyses, we find that liquid culture remains positive for longer than solid culture and smears assessed by FM staining are positive for longer than those assessed by ZN staining. This is perhaps expected, given liquid culture and FM staining are considered more sensitive detection methods.(23, 24) However, observed differences may also reflect imbalances in other covariates across subgroups. For example: more studies contributing to solid culture estimates, unlike those for liquid culture estimates, predated the HIV epidemic, whilst people living with HIV have been shown to have shorter culture conversion times.(20) Lack of disaggregation by conversion-predicting covariates and other key patient characteristics, such as HIV and the presence of cavitating lung disease, prevented any meaningful attempt to analyse the heterogeneity between and within subgroups. Additionally, point estimates of

times to conversion are expected to be influenced by the frequency of sampling. This parameter is often not reported.

We aimed to include only conversion data from patients receiving effective treatment. As the proportion of estimated new cases successfully treated for multidrug-resistant (MDR) TB globally was 55% at the time of this work,(1) we limited the review to patients receiving treatment for proven DS-TB. Our findings therefore cannot be extrapolated to infer the dynamics of smear and culture conversion in MDR-TB patients.

Many papers were excluded either because DST was not performed, or conversion estimates were not disaggregated by DST results. Adherence to therapy is also a critical component of effective treatment: many studies reported using directly observed therapy and some included other mechanisms for adherence support, but very few reported adherence data. Given the possibility of incomplete adherence, our analyses should be considered analogous to an intention-to-treat rather than an on-treatment protocol.

Beyond the indirect measure of sputum conversion times alone, we sought to explore when TB treatment renders patients non-infectious by reviewing studies using experimental animals as air samplers. In these studies, air extracted from isolation rooms housing TB patients is exhausted over susceptible guinea pigs, exposing them to infectious droplet nuclei. Modification of patient conditions, including the use of effective treatment, can be exploited to infer the effect of interventions on infectiousness, assessed by serial TST of exposed animals. These studies suggest that patients receiving effective TB treatment were less infectious than those not receiving such treatment, however, the temporal dynamics

infectiousness could not be elicited from the identified studies, individually or when combined.

This systematic review was performed to inform the WHO Global TB Department GDG of the evidence available to answer the question "How does the infectiousness of TB patients (ability to excrete viable bacteria and sustain transmission) change after having started effective TB treatment". Our summary estimates of culture conversion suggest that the majority of patients excrete viable bacilli at 2 weeks of treatment, and many continue to do so for several months. What this means for TB transmission and the two-week 'rule' cannot be determined by these analyses. A fuller understanding of the determinants of infectiousness of TB patients while on treatment necessitates a comprehensive evaluation of not only the persistence of viable *Mtb* in sputum, but also the contribution of other host and pathogen factors, including cough dynamics.

Acknowledgements

Particular thanks are extended to Maria Krutikov and Mengyun Liu for their assistance with duplicate sifting and data extraction, and the support from Lice González-Angulo and Fuad Mirzayev from WHO.

References

1. World Health Organisation. Global tuberculosis report 2019. Accessed from https://www.who.int/tb/publications/global_report/en/ (9th January 2020).
2. Fennelly KP, Jones-López EC, Ayakaka I, Kim S, Menyha H, Kirenga B, Muchwa C, Joloba M, Dryden-Peterson S, Reilly N, Okwera A, Elliott AM, Smith PG, Mugerwa RD, Eisenach KD, Ellner JJ. Variability of Infectious Aerosols Produced during Coughing by Patients with Pulmonary Tuberculosis. *Am J Respir Crit Care Med* 2012;doi:10.1164/rccm.201203-0444OC.
3. Jones-López EC, Namugga O, Mumbowa F, Ssebidandi M, Mbabazi O, Moine S, Mboowa G, Fox MP, Reilly N, Ayakaka I, Kim S, Okwera A, Joloba M, Fennelly KP. Cough aerosols of Mycobacterium tuberculosis predict new infection: A household contact study. *Am J Respir Crit Care Med* 2013;doi:10.1164/rccm.201208-1422OC.
4. Proaño A, Bravard MA, López JW, Lee GO, Bui D, Datta S, Comina G, Zimic M, Coronel J, Caviedes L, Cabrera JL, Salas A, Ticona E, Vu NM, Kirwan DE, Loader MCI, Friedland JS, Moore DAJ, Evans CA, Tracey BH, Gilman RH. Dynamics of cough frequency in adults undergoing treatment for pulmonary tuberculosis. *Clin Infect Dis* 2017;doi:10.1093/cid/cix039.
5. Rouillon A, Perdrizet S, Parrot R. Transmission of tubercle bacilli: The effects of chemotherapy. *Tubercle* 1976;doi:10.1016/S0041-3879(76)80006-2.
6. National Institute for Health and Care Excellence. Tuberculosis [NG33] (2016, updated 2019). Accessed from https://www.nice.org.uk/guidance/ng33 (9th January 2020).
7. Jensen PA, Lambert LA, Iademarco MF, Ridzon R, CDC. Guidelines for preventing the transmission of Mycobacterium tuberculosis in health-care settings, 2005. *MMWR Recomm reports Morb Mortal Wkly report Recomm reports* 2005;

8. Marchel Behr and Kevin Elwood. Canadian Tuberculosis Standards. *Public Heal Agency Canada* 2014;doi:10.1525/abt.2014.76.6.6.

9. Fitzwater SP, Caviedes L, Gilman RH, Coronel J, LaChira D, Salazar C, Saravia JC, Reddy K, Friedland JS, Moore DAJ. Prolonged Infectiousness of Tuberculosis Patients in a Directly Observed Therapy Short-Course Program with Standardized Therapy. *Clin Infect Dis* 2010;doi:10.1086/655127.

10. Loudon RG, Spohn SK. Cough frequency and infectivity in patients with pulmonary tuberculosis. *Am Rev Respir Dis* 1969;99:109–111.

11. World Health Organisation. WHO guidelines on tuberculosis infection prevention and control, 2019 update. 2019. Accessed from https://apps.who.int/iris/bitstream/handle/10665/311259/9789241550512-eng.pdf?ua=1 (9th January 2020).

12. Moher D, Liberati A, Tetzlaff J, Altman DG, Altman D, Antes G, Atkins D, Barbour V, Barrowman N, Berlin JA, Clark J, Clarke M, Cook D, D'Amico R, Deeks JJ, Devereaux PJ, Dickersin K, Egger M, Ernst E, Gøtzsche PC, Grimshaw J, Guyatt G, Higgins J, Ioannidis JPA, Kleijnen J, Lang T, Magrini N, McNamee D, Moja L, *et al.* Preferred reporting items for systematic reviews and meta-analyses: The PRISMA statement. *PLoS Med* 2009;doi:10.1371/journal.pmed.1000097.

13. World Health Organisation. Guidelines for treatment of drug-susceptible tuberculosis and patient care, 2017 update. 2017. Accessed from https://www.who.int/tb/publications/2017/dstb_guidance_2017/en/ (9th January 2020).

14. National Institutes of Health. Study Quality Assessment Tools: Quality Assessment Tool for Case Series Studies. Accessed from https://www.nhlbi.nih.gov/health-

topics/study-quality-assessment-tools (9th January 2020).

15. Freeman MF, Tukey JW. Transformations Related to the Angular and the Square Root. *Ann Math Stat* 2007;doi:10.1214/aoms/1177729756.

16. Schwarzer G. Meta-analysis with R. Version 1.5. Available at rdocumentation.org/packages/meta/versions/1.5-0.

17. Riley RL, Mills CC, O'Grady F, Sultan LU, Wittstadt F, Shivpuri DN. Infectiousness of air from a tuberculosis ward. Ultraviolet irradiation of infected air: comparative infectiousness of different patients. *Am Rev Respir Dis* 1962;

18. Escombe AR, Oeser C, Gilman RH, Navincopa M, Ticona E, Martinez C, Caviedes L, Sheen P, Gonzalez A, Noakes C, Moore DAJ, Friedland JS, Evans CA. The Detection of Airborne Transmission of Tuberculosis from HIV-Infected Patients, Using an In Vivo Air Sampling Model. *Clin Infect Dis* 2007;doi:10.1086/515397.

19. Escombe AR, Moore DAJ, Gilman RH, Pan W, Navincopa M, Ticona E, Martínez C, Caviedes L, Sheen P, Gonzalez A, Noakes CJ, Friedland JS, Evans CA. The infectiousness of tuberculosis patients coinfected with HIV. *PLoS Med* 2008;doi:10.1371/journal.pmed.0050188.

20. Dharmadhikari AS, Mphahlele M, Venter K, Stoltz A, Mathebula R, Masotla T, Van Der Walt M, Pagano M, Jensen P, Nardell E. Rapid impact of effective treatment on transmission of multidrug-resistant tuberculosis. *Int J Tuberc Lung Dis* 2014;doi:10.5588/ijtld.13.0834.

21. Telzak EE, Fazal BA, Pollard CL, Turett GS, Justman JE, Blum S. Factors influencing time to sputum conversion among patients with smear-positive pulmonary tuberculosis. *Clin Infect Dis* 1997;25:666–70.

22. Domínguez-Castellano A, Muniain MA, Rodriguez-Baño J, Garcia M, Rios MJ, Galvez J,

Perez-Cano R. Factors associated with time to sputum smear conversion in active pulmonary tuberculosis. *Int J Tuberc Lung Dis* 2003;

23. Asmar S, Drancourt M. Rapid culture-based diagnosis of pulmonary tuberculosis in developed and developing countries. *Front Microbiol* 2015;doi:10.3389/fmicb.2015.01184.

24. Steingart KR, Henry M, Ng V, Hopewell PC, Ramsay A, Cunningham J, Urbanczik R, Perkins M, Aziz MA, Pai M. Fluorescence versus conventional sputum smear microscopy for tuberculosis: a systematic review. *Lancet Infect Dis* 2006;doi:10.1016/S1473-3099(06)70578-3.

25. Abal AT, Jayakrishnan B, Parwer S, El Shamy A, Abahussain E, Sharma PN. Effect of cigarette smoking on sputum smear conversion in adults with active pulmonary tuberculosis. *Respir Med* 2005;doi:10.1016/j.rmed.2004.08.016.

26. Chaulet P, Boulahbal FCN-FC. [Clinical trial of a combination of three drugs in fixed proportions in the treatment of tuberculosis. Groupe de Travail sur la Chimiothérapie de la Tuberculose]. *Int Union against Tuberc Lung Dis* 1995;

27. Conde MB, Efron A, Loredo C, De Souza GRM, Graça NP, Cezar MC, Ram M, Chaudhary MA, Bishai WR, Kritski AL, Chaisson RE. Moxifloxacin versus ethambutol in the initial treatment of tuberculosis: a double-blind, randomised, controlled phase II trial. *Lancet* 2009;doi:10.1016/S0140-6736(09)60333-0.

28. Conde MB, Mello FCQ, Duarte RS, Cavalcante SC, Rolla V, Dalcolmo M, Loredo C, Durovni B, Armstrong DT, Efron A, Barnes GL, Marzinke MA, Savic RM, Dooley KE, Cohn S, Moulton LH, Chaisson RE, Dorman SE. A phase 2 randomized trial of a rifapentine plus moxifloxacin-based regimen for treatment of pulmonary tuberculosis. *PLoS One* 2016;doi:10.1371/journal.pone.0154778.

29. Dawson R, Condos R, Tse D, Huie ML, Ress S, Tseng CH, Brauns C, Weiden M, Hoshino Y, Bateman E, Rom WN. Immunomodulation with recombinant interferon-γ1b in pulmonary tuberculosis. *PLoS One* 2009;doi:10.1371/journal.pone.0006984.

30. Dawson R, Diacon AH, Everitt D, Van Niekerk C, Donald PR, Burger DA, Schall R, Spigelman M, Conradie A, Eisenach K, Venter A, Ive P, Page-Shipp L, Variava E, Reither K, Ntinginya NE, Pym A, Von Groote-Bidlingmaier F, Mendel CM. Efficiency and safety of the combination of moxifloxacin, pretomanid (PA-824), and pyrazinamide during the first 8 weeks of antituberculosis treatment: A phase 2b, open-label, partly randomised trial in patients with drug-susceptible or drug-resistant pul. *Lancet* 2015;doi:10.1016/S0140-6736(14)62002-X.

31. Dawson R, Narunsky K, Carman D, Gupte N, Whitelaw A, Efron A, Barnes GL, Hoffman J, Chaisson RE, McIlleron H, Dorman SE. Two-stage activity-safety study of daily rifapentine during intensive phase treatment of pulmonary tuberculosis. *Int J Tuberc Lung Dis* 2015;doi:10.5588/ijtld.14.0868.

32. Desjardin LE, Perkins MD, Wolski K, Haun S, Teixeira L, Chen Y, Johnson JL, Ellner JJ, Dietze R, Bates J, Cave MD, Eisenach KD. Measurement of sputum Mycobacterium tuberculosis messenger RNA as a surrogate for response to chemotherapy. *Int J Lepr Other Mycobact Dis* 1999;

33. Dlugovitzky D, Fiorenza G, Farroni M, Bogue C, Stanford C, Stanford J. Immunological consequences of three doses of heat-killed Mycobacterium vaccae in the immunotherapy of tuberculosis. *Respir Med* 2006;doi:10.1016/j.rmed.2005.09.026.

34. Dorman SE, Johnson JL, Goldberg S, Muzanye G, Padayatchi N, Bozeman L, Heilig CM, Bernardo J, Choudhri S, Grosset JH, Guy E, Guyadeen P, Leus MC, Maltas G, Menzies D, Nuermberger EL, Villarino M, Vernon A, Chaisson RE, Mugerwa RD, Mayanja-Kizza

H, Gitta P, Okwera A, Lamunu D, Nsubuga P, Joloba M, Morgan K, Mulumba Y, Nakibali JG, *et al.* Substitution of moxifloxacin for isoniazid during intensive phase treatment of pulmonary tuberculosis. *Am J Respir Crit Care Med* 2009;doi:10.1164/rccm.200901-0078OC.

35. Dorman SE, Goldberg S, Stout JE, Muzanyi G, Johnson JL, Weiner M, Bozeman L, Heilig CM, Feng PJ, Moro R, Narita M, Nahid P, Ray S, Bates E, Haile B, Nuermberger EL, Vernon A, Schluger NW. Substitution of rifapentine for rifampin during intensive phase treatment of pulmonary tuberculosis: Study 29 of the tuberculosis trials consortium. *J Infect Dis* 2012. doi:10.1093/infdis/jis461.

36. Dorman SE, Savic RM, Goldberg S, Stout JE, Schluger N, Muzanyi G, Johnson JL, Nahid P, Hecker EJ, Heilig CM, Bozeman L, Feng PJI, Moro RN, MacKenzie W, Dooley KE, Nuermberger EL, Vernon A, Weiner M. Daily rifapentine for treatment of pulmonary tuberculosis: A randomized, dose-ranging trial. *Am J Respir Crit Care Med* 2015;doi:10.1164/rccm.201410-1843OC.

37. East and Central African / British Medical Research Council. Controlled clinical trial of 4 short-course regimens of chemotherapy (three 6-month and one 8-month) for pulmonary tuberculosis. *Tubercle* 1983;doi:10.1016/0041-3879(83)90011-9.

38. Grandjean L, Gilman RH, Martin L, Soto E, Castro B, Lopez S, Coronel J, Castillo E, Alarcon V, Lopez V, San Miguel A, Quispe N, Asencios L, Dye C, Moore DAJ. Transmission of Multidrug-Resistant and Drug-Susceptible Tuberculosis within Households: A Prospective Cohort Study. *PLoS Med* 2015;doi:10.1371/journal.pmed.1001843.

39. Hong Kong Chest Service / British Medical Research Council. Controlled trial of four thrice-weekly regimens and a daily regimen all given for six months for pulmonary

tuberculosis. *Lancet* 1981;doi:10.1016/S0140-6736(81)90057-X.

40. Hong Kong Chest Service / British Medical Research Council. Controlled trial of 6-month and 8-month regimens in the treatment of pulmonary tuberculosis. *Am Rev Respir Dis* 1978;118:219–28.

41. Jindani A, Harrison TS, Nunn AJ, Phillips PPJ, Churchyard GJ, Charalambous S, Hatherill M, Geldenhuys H, McIlleron HM, Zvada SP, Mungofa S, Shah NA, Zizhou S, Magweta L, Shepherd J, Nyirenda S, Van Dijk JH, Clouting HE, Coleman D, Bateson ALE, McHugh TD, Butcher PD, Mitchison DA. High-dose rifapentine with moxifloxacin for pulmonary tuberculosis. *N Engl J Med* 2014;doi:10.1056/NEJMoa1314210.

42. Jindani A, Borgulya G, Westermann De Patino I, Gonzales T, De Fernandes RA, Shrestha B, Atwine D, Bonnet M, Burgos M, Dubash F, Patel N, Checkley AM, Harrison TS, Mitchison D. A randomised Phase II trial to evaluate the toxicity of high-dose rifampicin to treat pulmonary tuberculosis. *Int J Tuberc Lung Dis* 2016;doi:10.5588/ijtld.15.0577.

43. Johnson JL, Kamya RM, Okwera A, Loughlin AM, Nyole S, Hom DL, Wallis RS, Hirsch CS, Wolski K, Foulds J, Mugerwa RD, Ellner JJ. Randomized Controlled Trial of Mycobacterium vaccae Immunotherapy in Non–Human Immunodeficiency Virus–Infected Ugandan Adults with Newly Diagnosed Pulmonary Tuberculosis . *J Infect Dis* 2000;doi:10.1086/315393.

44. Johnson JL, Ssekasanvu E, Okwera A, Mayanja H, Hirsch CS, Nakibali JG, Jankus DD, Eisenach KD, Boom WH, Ellner JJ, Mugerwa RD. Randomized trial of adjunctive interleukin-2 in adults with pulmonary tuberculosis. *Am J Respir Crit Care Med* 2003;doi:10.1164/rccm.200211-1359OC.

45. Joloba ML, Johnson JL, Namale A, Morrissey A, Assegghai AE, Mugerwa RD, Ellner JJ,

Eisenach KD. Quantitative sputum bacillary load during rifampin-containing short course chemotherapy in human immunodeficiency virus-infected and non-infected adults with pulmonary tuberculosis. *Int J Tuberc Lung Dis* 2000;

46. Kanda R, Nagao T, Van Tho N, Ogawa E, Murakami Y, Osawa M, Saika Y, Doi K, Nakano Y. Factors affecting time to sputum culture conversion in adults with pulmonary tuberculosis: A historical cohort study without censored cases. *PLoS One* 2015;doi:10.1371/journal.pone.0142607.
47. Kennedy N, Berger L, Curram J, Fox R, Gutmann J, Kisyombe GM, Ngowi FI, Ramsay ARC, Saruni AOS, Sam N, Tillotson G, Uiso LO, Yates M, Gillespie SH. Randomized controlled trial of a drug regimen that includes ciprofloxacin for the treatment of pulmonary tuberculosis. *Clin Infect Dis* 1996;doi:10.1093/clinids/22.5.827.
48. Long R, Bochar K, Chomyc S, Talbot J, Barrie J, Kunimoto D, Tilley P. Relative Versus Absolute Noncontagiousness of Respiratory Tuberculosis on Treatment. *Infect Control Hosp Epidemiol* 2003;doi:10.1086/502145.
49. Méchaï F, Figoni J, Leblanc C, Gousseff M, Vignier N, Bouchaud O. Active pulmonary tuberculosis: Role for amikacin in early treatment. *Médecine Mal Infect* 2016;doi:10.1016/j.medmal.2016.04.010.
50. Pheiffer C, Carroll NM, Beyers N, Donald P, Duncan K, Uys P, Van Helden P. Time to detection of Mycobacterium tuberculosis in BACTEC systems as a viable alternative to colony counting. *Int J Tuberc Lung Dis* 2008;
51. Rathored J, Sharma SK, Singh B, Banavaliker JN, Sreenivas V, Srivastava AK, Mohan A, Sachan A, Harinarayan C V., Goswami R. Risk and outcome of multidrug-resistant tuberculosis: Vitamin D receptor polymorphisms and serum 25(OH)D. *Int J Tuberc Lung Dis* 2012;doi:10.5588/ijtld.12.0122.

52. Singapore Tuberculosis Service / British Medical Research Council. Clinical trial of 6-month and 4-month regimens of chemotherapy in the treatment of pulmonary tuberculosis. *AmerRevRespDis* 1979;119:579–85.

53. Singapore Tuberculosis Service / British Medical Research Council. Clinical trial of three 6-month regimens of chemotherapy given intermittently in the continuation phase in the treatment of pulmonary tuberculosis. *Am Rev Respir Dis1* 1985;132:374–8.

54. Singla R, Osman MM, Khan N, Al-Sharif N, Al-Sayegh MO, Shaikh MA. Factors predicting persistent sputum smear positivity among pulmonary tuberculosis patients 2 months after treatment. *Int J Tuberc Lung Dis* 2003;

55. Stoffel C, Imaz MS, Lorenz R, Arce M, Fernández L, Rico M. Treatment of pulmonary tuberculosis in a low-prevalence urban area. Compliance and sputum conversion. *Medicina (B Aires)* 2014;

56. Tanzania / British Medical Research Council. Controlled clinical trial of two 6-month regimens of chemotherapy in the treatment of pulmonary tuberculosis. *Am Rev Respir Dis1* 1985;131:727–31.

57. Tanzania / British Medical Research Council. A controlled trial of a 4-weekly supplement of rifampicin, pyrazinamide and streptomycin in the continuation phase of a 7-month daily chemotherapy regimen for pulmonary tuberculosis. *South African Med J* 1996;

58. British Thoracic Association. A controlled trial of six months chemotherapy in pulmonary tuberculosis. First report: Results during chemotherapy. *Br J Dis Chest* 1981;75:141–53.

59. Combs DL, O’Brien RJ, Geiter LJ. USPHS tuberculosis short-course chemotherapy trial

21: Effectiveness, toxicity, and acceptability. The report of final results. *Ann Intern Med* 1990;doi:10.7326/0003-4819-76-3-112-6-397.

60. Lee HY, Chae KO, Lee CH, Choi SM, Lee J, Park YS, Lee SM, Yoo CG, Kim YW, Han SK, Yim JJ. Culture conversion rate at 2 months of treatment according to diagnostic methods among patients with culture-positive pulmonary tuberculosis. *PLoS One* 2014;doi:10.1371/journal.pone.0103768.

61. Leung CC, Yew WW, Mok TYW, Lau KS, Wong CF, Chau CH, Chan CK, Chang KC, Tam G, Tam CM. Effects of diabetes mellitus on the clinical presentation and treatment response in tuberculosis. *Respirology* 2017;doi:10.1111/resp.13017.

62. Musteikienė G, Miliauskas S, Zaveckienė J, Žemaitis M, Vitkauskienė A. Factors associated with sputum culture conversion in patients with pulmonary tuberculosis. *Med* 2017;doi:10.1016/j.medici.2018.01.005.

63. Sajid MUD, Imran M, Siddiqique M. Addition of amikacin and levofloxacin is associated with higher culture conversion rate in pulmonary tuberculosis. *Pakistan J Med Heal Sci* 2011;

64. Scott C, Cavanaugh JS, Silk BJ, Ershova J, Mazurek GH, LoBue PA, Moonan PK. Comparison of sputum-culture conversion for Mycobacterium bovis and M. Tuberculosis. *Emerg Infect Dis* 2017;doi:10.3201/eid2303.161916.

65. Tuberculosis Research Centre. Study of chemotherapy regimens of 5 and 7 months' duration and the role of corticosteroids in the treatment of sputum-positive patients with pulmonary tuberculosis in South India. *Tubercle* 1983;doi:10.1016/0041-3879(83)90032-6.

66. Volkmann T, Moonan PK, Miramontes R, Oeltmann JE. Tuberculosis and excess alcohol use in the United States, 1997-2012. *Int J Tuberc Lung Dis*

2015;doi:10.5588/ijtld.14.0516.

67. Click ES, Winston CA, Oeltmann JE, Moonan PK, Mac Kenzie WR. Association between Mycobacterium tuberculosis lineage and time to sputum culture conversion. *Int J Tuberc Lung Dis* 2013;doi:10.5588/ijtld.12.0732.

68. Salindri AD, Sales RMF, DiMiceli L, Schechter MC, Kempker RR, Magee MJ. Isoniazid monoresistance and rate of culture conversion among patients in the state of Georgia with confirmed tuberculosis, 2009-2014. *Ann Am Thorac Soc* 2018;doi:10.1513/AnnalsATS.201702-147OC.

Figure Legends

Figure 1. Flow diagram showing databases searched, records found, sifted, and included, and reasons for exclusion at full text review.

Figure 2. Proportion of baseline smear-positive patients receiving effective treatment for drug-susceptible TB achieving smear conversion at 2, 3 weeks and 1, 2, 3 and 4 months. CI: confidence interval; ES: summary estimate; TB: tuberculosis. BMRC: British Medical Research Council; ECA: East and Central African.

Figure 3. Proportion of baseline culture-positive patients receiving effective treatment for drug-susceptible TB achieving solid-culture conversion at 1, 2 and 3 weeks, and at 1, 2, 3 and 4 months. CI: confidence interval; ES: summary estimate; TB: tuberculosis; BTA: British Thoracic Association; TBRC: Tuberculosis Research Centre; STBS: Singapore Tuberculosis Service; BMRC: British Medical Research Council; T: Tanzania; HKCS: Hong Kong Chest Service.

Figure 4. Proportion of baseline culture-positive patients receiving effective treatment for drug-susceptible TB achieving liquid culture conversion at 2 and 3 weeks, and at 1, 2, 3, and 4 months. CI: confidence interval; ES: summary estimate; TB: tuberculosis.

Figure 5. Proportion of baseline smear-positive patients receiving effective treatment for drug-susceptible TB, achieving solid culture and liquid culture conversion at 2 weeks and 2 months. CI: confidence interval; ES: summary estimate; T: Tanzania; ECA: East and Central Africa; BMRC: British Medical Research Council; HKCS: Hong Kong Chest Services; STBS: Singapore Tuberculosis Service.

Figure 6. Flow diagram showing the animal studies identified for review.

Footnotes

Figure 1.
Abbreviations:
N = number of studies; RCT = randomised controlled trials
Footnotes: Two searches were developed conducted by a professional librarian, a primary search and a second search which aimed to identify all randomised controlled trials of tuberculosis treatment conducted since 1990, as described in text.[None]

Figure 2.
Abbreviations: CI = confidence interval; BMRC = British Medical Research Council; ECA = East and Central African.

Figure 3.
Abbreviations: CI = confidence interval; BTA = British Thoracic Association; TBRC = Tuberculosis Research Centre; STBS = Singapore Tuberculosis Service; BMRC = British Medical Research Council; T = Tanzania; HKCS = Hong Kong Chest Service.

Figure 4.
Abbreviations: CI = confidence interval

Figure 5.
Abbreviations: CI = confidence interval; T = Tanzania; ECA = East and Central Africa; BMRC = British Medical Research Council; HKCS = Hong Kong Chest Services; STBS = Singapore Tuberculosis Service.

Figure 6.
Abbreviations: n = number of studies

Tables

Table 1. Summary of inclusion and exclusion criteria employed during record sifting		
	**Inclusion criteria**	**Exclusion criteria**
**Participants**	1. Bacteriologically confirmed (smear or culture-positive) PTB<br>2. Confirmed drug-susceptibility to, at least, rifampicin and isoniazid*<br>3. Treated with a regimen including rifampicin and isoniazid with pyrazinamide added during two-month 'intensive phase', administered at least five days per week†	1. Any study in a not reporting data on participants with confirmed, drug-susceptible PTB (or disaggregated data for this subgroup).<br>2. Any study not in humans<br>3. Any study not reporting on participants treated with a conventional first line regimen(13) (e.g. addition of quinolones), or treatment given less than five days per week†
**Outcome measures**	Studies reporting data on at least one of the outcome measures of interest:<br>4. Proportion of participants achieving sputum smear microscopy or culture conversion‡, assessed at any time-point within the first four months of treatment.<br>5. A summary measure (such as mean or median) of time to conversion, assessed by repeated smear or culture‡ assessment (at any interval) during treatment	4. Any study not reporting on any of the above-stated outcomes of interest
**Study types**	6. Any consecutive case series, case control study, cohort study, randomised controlled study, systematic review, or meta-analysis.	5. Any systematic review superseded by an updated systematic review<br>6. Narrative reviews not adding new data or new analysis of data to existing knowledge<br>7. Commentaries and mathematical modelling studies<br>8. Studies with fewer than 10 participants per arm<br>9. Any study not written in English, Japanese, Chinese, Russian, French, Spanish, or Portuguese<br>10. Any study published before 1946

Footnotes:
* Criterion only applied at full text review as infrequently reported in abstracts
† Where studies used an included regimen for an initial period, with an excluded regimen later (e.g. during continuation phase, or insufficient duration of pyrazinamide), the study was included and data extracted only for the period where an included regimen was administered
‡ Sputum smear and culture assessment could be by any method including Ziehl-Neelsen stain, fluorescence microscopy, liquid and solid culture, or by undefined culture method.

Table 2. Description of included studies

Author	Year	Location	Study design	N assessed[*]	Age[†]	% Female	% HIV+	Treatment[‡] Intensive	Continuation	Adherence	Outcome reported	Assessment method[§]	Study dates
**Smear positive pulmonary TB**													
Abal(25)	2005	Kuwait	C (p)	339	-	Smoker-: 42%<br>Smoker+: 1%	-	2RHZ±E/S	-	-	PC	Smear (ZN)	1998 – 2000
Chaulet(26)	1995	Algeria	RCT	196	-	26%	-	2RHZ	4RH	IP DOT (≥14d) / U-INH	PC	Culture (NS)	-
Conde(27)	2009	Brazil	RCT	72	*36 (± 12)*	31%	3%	2RHZE (5d)	4RH	DOT	Both	Culture (Solid)	2004 – 2007
Conde(28)	2016	Brazil	RCT	45 [59]	30 (24-45)	39%	0%	2RHZE	4RH	DOT	Both	Culture (Solid + Liquid)	2009 – 2013
Dawson(29)	2009	South Africa	RCT	30	32 (± 11)	37%	7%	2RHZE	4RH[¶]	DOT	PC	Smear (NS)	2005 – 2006
Dawson(30)	2015 a	South Africa, Tanzania	RCT	54 [59]	*30.4 (± 10)*	31%	22%	2RHZE	-	-	Both	Culture (Solid + Liquid)	2012 – 2013
Dawson(31)	2015 b	South Africa	RCT	36 [48]	30 (25-36)	27%	6%	2RHZE	4RH	DOT[||]	Both	Culture (Solid + Liquid)	2010 – 2013
DesJardin(32)	1999	Brazil	RCT	19	*34 (range 19-55)*	26%	0%	2RHZE	-	DOT (≥2w) / pill count / U-INH	PC	Culture (Solid)	-
Dlugovitzky(33)	2006	Argentina	RCT	10	*37 (± 13)*	20%	0%	2RHZE	4RH	DOT	PC	Smear (ZN) Culture (Solid)	≥ 2001
Dominguez-Castellano(22)	2003	Spain	C (p)	95 [109]	-	-	39%	2RHZ±E	NS	-	TTC	Smear (ZN)	-
Dorman(34)	2009	Multiple (4 continents)	RCT	164	30 (25-40)	28%	11%	2RHZE (5d)	-	DOT[||]	PC	Culture (Solid + Liquid)	2006 – 2007
Dorman(35)	2012	Multiple (4 continents)	RCT	183	34 (26-47)	37%	13%	2RHZE (5d)	-	DOT[||]	PC	Culture (Solid + Liquid)	2008 – 2010
Dorman(36)	2015	Multiple (4 continents)	RCT	64 [85]	33 (19-78)	35%	6%	2RHZE	-	DOT	PC	Culture (Solid + Liquid)	2011 – 2012
E&C Africa / BMRC(37)	1983	Multiple (SSA)	RCT	708	-	35%	-	2RHZS	-	IP DOT	PC	Smear (NS)	1978 – 1980
Grandjean(38)	2015	Peru	C (p)	487	29	61%	4%	2RHZE[¶]	-	-	PC	Smear (NS)	2010 – 2013
HKCS / BMRC(39)	1981	Hong Kong	RCT	168 [1207]	-	28%	-	6RHZE	-	DOT[||]	PC	Culture (Solid)	-
HKCS / BMRC(40)	1978	Hong Kong	RCT	180 [842]	-	29%	-	2RHZS	-	DOT	PC	Culture (Solid)	-
Jindani(41)	2014	Multiple (SSA)	RCT	163	-	36%	29%	2RHZE	-	DOT	PC	Culture (Solid + Liquid)	2008 – 2011
Jindani(42)	2016	Multiple (SSA, S. Am)	RCT	92 [100]	29 (range 18-67)	34%	0%	2RHZE	-	DOT	PC	Culture (Solid)	2011 – 2013
Johnson(43)	2000	Uganda	RCT	52 [59]	*29 (± 8)*	22%	0%	2RHZE	4RH	pill count / U-INH	PC	Culture (Solid)	1995 – 1997
Johnson(44)	2003	Uganda	RCT	47 [55]	*27.4 (± 7)*	25%	0%	2RHZE	-	IP DOT (≥1m) / U-INH	PC	Culture (Solid)	-

Joloba(45)	2000	Uganda	C (p)	36	*HIV-: 31 (±9)*<br>*HIV+: 27 (±8)*	HIV-: 57%<br>HIV+: 50%	61%	2RHZE	-	DOT (≥2w)	PC	Culture<br>(Solid + Liquid)	1996 – 1997
Kanda(46)	2015	Japan	C (r)	86	57<br>(range 40-67)	30%	0%	2RHZ+E/S	≥4RH	IP DOT	Both	Culture (Solid)	-
Kennedy(47)	1996	Tanzania	RCT	86	*36 (± 13)*	36%	37%	2RHZE	2RHZ+2RH	IP DOT	TTC	Smear (FM)<br>Culture (NS)	1990 – 1992
Long(48)	2003	Canada	C (p/r)	32	-	38%	0%	2RHZ	-	DOT[||]	Both	Smear (ZN)	1997 – 1999
Mechai(49)	2016	France	C (p)	30	43 (36-52)	23%	0%	2RHZE	4RH	-	TTC	Smear (NS)<br>Culture (NS)	2009 – 2013
Pheiffer(50)	2008	South Africa	C (p)	105	-	-	-	2RHZE	-	-	PC	Smear (ZN)	2000 – 2004
Rathored(51)	2012	India	C (p)	50 [338]	*27 (± 10)*	28%	0%	2RHZE[¶]	2RH[¶]	DOT	TTC	Smear (ZN)<br>Culture (Solid)	2006 – 2011
STBS / BMRC(52)	1979	Singapore	RCT	330 [363]	-	35%	-	2RHZS	-	DOT	PC	Culture (Solid)	-
STBS / BMRC(53)	1985	Singapore	RCT	319	-	40%	-	1/2HRZ±E	-	DOT	PC	Culture (Solid)	-
Singla(54)	2003	Saudi Arabia	C (r)	434 [514]	*M: 38 (±33)*<br>*F: 33 (±15)*	37%	0%	2RHZE	-	IP DOT (≥1m)	PC	Smear (NS)	1998 – 1999
Stoffel(55)	2014	Argentina	C (r)	148	-	34%	-	2RHZE	-	DOT	PC	Smear (ZN)<br>Culture (Solid)	2000 – 2010
Tanzania / BMRC(56)	1985	Tanzania	RCT	224	-	29%	-	2RHZS	-	IP DOT / U-INH	PC	Culture (Solid)	1978 – 1981
Tanzania / BMRC(57)	1996	Tanzania	RCT	266	-	31%	-	1.5RHZS	-	IP DOT / U-INH	PC	Culture (NS)	1982 – 1985
Telzak(21)	1997	USA	C (r)	77 [100]	-	39%	59%	RHZE[¶**]	RH[||]	DOT	TTC	Smear (ZN)<br>Culture (NS)	1993 – 1996
**Any pulmonary TB**													
BTA(58)	1981	UK	RCT	287<br>[334]	*RHZS: 38*<br>*RHZE: 39*	RHZS:33%<br>RHZE:38%	-	RHZ+E/S	4RH	U-INH	PC	Culture (Solid)	-
Combs(59)	1990	USA	RCT	617	*41 (± 15)*	22%	-	2RHZ±E	4RH	-	PC	Culture (NS)	1981 - 1986
Lee(60)	2014	South Korea	C (r)	162	S+: 58 (43-70)<br>C+ 58 (40-70)	S+: 38%<br>C+: 42%	<1%	2RHZE[¶]	-	-	Both	Culture<br>(Liquid)	2011 – 2012
Leung(61)	2017	China (Hong Kong)	C (p)	15658<br>[21414]	*54 (± 21)*	36%	<1%	≥RHZ	NS	-	PC	Smear (NS)<br>Culture (NS)	2006 – 2010
Musteikiene(62)	2017	Lithuania	C (p)	52	-	77%	0%	2RHZE	4RH	IP DOT	PC	Culture (Solid)	2015 – 2016
Sajid(63)	2011	Pakistan	RCT	50	*49 (± 18)*	38%	0%	2RHZE	-	-	PC	Culture (NS)	2009 – 2010
Scott(64) [††]	2017	USA	C (r)	30848	46 (30-60)	34%	6%[‡‡]	2RHZE[¶**]	2RH[¶]	-	PC	Culture (NS)	2006 – 2013
TBRC(65)	1983	India	RCT	261 [390]	-	25%	-	2RHZS	-	DOT[||]	PC	Culture (Solid)	-
Volkmann(66) [††]	2015	USA	C (r)	60034<br>[207307]	-	38%	8%	2RHZE[¶]	2RH[¶]	DOT (54%[§])	Both	Culture (NS)	1997 – 2012

Abbreviations

BMRC = British Medical Research Council
BTA = British Thoracic Association
C = cohort study (p = prospective, r = retrospective)
C+ = culture positive subgroup
DOT = directly observed therapy (IP = as hospital inpatient; values in brackets indicate duration with d = days, w = weeks, m = months)

E = Ethambutol
E&C = East & Central
H = Isoniazid
HIV-/+ = people living without/with HIV respectively
HKCS = Hong Kong Chest Service
N assessed = number assessed for outcome.
NS = not specified
PC = proportion converted at a timepoint
R = Rifampicin
RCT = randomised controlled trial
S+ = smear positive subgroup
STBS = Singapore Tuberculosis Service
Smoker-/+ = without / with smoking exposure respectively
TBRC = Tuberculosis Research Centre
TTC =summary measure of time to conversion
U-INH = urine isoniazid metabolite testing
Z = pyrazinamide
- = data not available

Footnotes
***** Where demographics were not given for the population assessed for outcome, number in brackets indicates the number for which demographics are stated (which may include DRTB, non-HRZ containing regimens, etc.) Where available, per-protocol or efficacy analyses were extracted.
† Age is given as median (interquartile range) or (italics) *mean (± standard deviation)* unless otherwise stated.
‡ Treatment given seven days per week unless otherwise stated (5/7 = 5 days per week).
§ For studies performed before 1990, culture type assumed to be solid unless otherwise stated.
|| Individuals not adherent to DOT (as defined by authors) excluded from analysis.
¶ Paper stated treatment was in accordance with National or International guidelines; corresponding regimen shown here. In all others, drugs used were explicitly stated.
** It is not possible to exclude that some patients were treated with thrice-weekly regimens in these studies.
†† These studies use the same dataset and overlapping time frames; however, the estimates presented are different. They therefore contribute to different analyses. Two further papers which include overlapping data were excluded to avoid duplication.(67, 68)
‡‡ Includes a proportion of patients with unknown HIV status.
§§ Percentage of study population receiving DOT

Table 3. Number of estimates contributing to proportion converted analysis by time point and microbiological assessment method

Duration of treatment	Smear	Culture			Total
		Solid	Liquid	Not specified	
**1 week**	0	4	0	0	4
**2 weeks**	1	3	1	0	5
**3 weeks**	1	3	1	1	6
**1 month**	4	12	2	2	20
**2 months**	10	23	9	4	46
**3 months**	1	3	1	2	7
**4 months**	1	5	1	1	8
**Total**	**18**	**53**	**15**	**10**	**96**

Table 4. Studies reporting a summary measure of time to smear conversion*								
**First author**	**Year**	**Country**	**N**	**Sampling frequency**	**Smear stain**	**Median, days**	**Mean, days**	**Spread (method)**
Kennedy(47)	1996	Tanzania	81	monthly	FM	-	55	30–120 (range)
Dominguez-Castellano(22)	2003	Spain	95	weekly	ZN	20	-	± 2 (SE)
Telzak(21)	1997	USA	77	weekly	ZN	-	33	± 6.2 (SE)
Long(48)	2003	Canada	32	daily to day 14 then x2/week†	FM	-	46	± 27.9 (SD)
Rathored(51)	2012	India	50	weekly	ZN	-	29	± 0.7 (SD)
Mechai(49)	2016	France	30	x2/week‡	NS	27	-	14-56 (IQR)

Abbreviations:
FM = fluorescence microscopy;
IQR – interquartile range;
N = number assessed for outcome
NS = not stated;
SD = standard deviation;
SE = standard error;
ZN = Ziehl-Neelsen

Footnotes:
* Where authors reported data in months these have been converted to days to aid comparisons (using 30.4 days per month).
† for 14 prospectively studied patients; for 18 retrospectively studies patients sampling was weekly (mean of 1.6 per week) throughout treatment;
‡ on days 7-9 then on 3 days every two weeks.

Table 5. Studies reporting a summary measure of time to solid or liquid culture conversion*

First author	Year	Country	Smear+	Sampling frequency	Solid culture			Liquid culture	
					N	Median, days (IQR)	Mean, days (SD/SE)	N	Median, days (IQR)
Conde(27)	2009	Brazil	100%	weekly	72	49	-		
Rathored (51)	2012	India	100%	every 2 weeks	50	-	24 ± 0.7 (SD)		
Lee(60)	2014	South Korea	100%<br>0%	monthly after 2 weeks				61<br>101	40 (28-61)<br>19 (1-41)
Kanda(46)	2015	Japan	100%	every 2 weeks	86	39 (25-55)	-		
Dawson(30)	2015a	South Africa, Tanzania	100%	weekly	54	35	-	56	56
Dawson(31)	2015b	South Africa	100%	weekly	36	43 (29-52)	46 ± 27.9 (SD)	38	59 (36-63)
Conde(28)	2016	Brazil	100%	weekly	45	41 (35-48)		36	52 (41-59)

Abbreviations:
N = number assessed for outcome
NS = not stated;
IQR = interquartile range;
SD = standard deviation;
SE = standard error;
USA = United States of America;
- = data not available

Footnotes:
* Where authors reported data in months these have been converted to days to aid comparisons using 30.4 days per month.

Table 6. Overview of summary estimates derived from random effects meta-analysis for the proportion of patients achieving culture conversion at each time point, by culture detection method

Duration of treatment	Smear microscopy			Solid culture			Liquid culture		
	Proportion (95% CI)	N	I^2	Proportion (95% CI)	N	I^2	Proportion (95% CI)	N	I^2
1 week	-	0	-	0.01 (0.00-0.03)	4	44%	-	0	-
2 weeks	0.09 (0.03-0.24)	1	-	0.10 (0.01-0.26)	3	-	0.03 (0.01-0.16)	1	-
3 weeks	0.25 (0.13-0.42)	1	-	0.22 (0.19-0.25)	3	-	0.06 (0.02-0.20)	1	-
1 month	0.33 (0.25-0.42)	4	32%	0.46 (0.39-0.54)	12	91%	0.17 (0.09-0.25)	1	-
2 months	0.82 (0.78-0.86)	10	89%	0.86 (0.81-0.91)	23	93%	0.63 (0.50-0.75)	9	92%
3 months	0.94 (0.94-0.95)	1	-	0.94 (0.72-1.00)	3	-	0.56 (0.39-0.71)	1	-
4 months	1.00 (0.72-1.00)	1	-	0.97 (0.82-1.00)	5	97%	0.82 (0.64-0.92)	1	-

Abbreviations:
CI = confidence interval;
N = number of estimates;
I^2 = I^2 test for heterogeneity

Table 7. Studies evaluated for data on TB patient infectiousness to animals during TB treatment.

First author	Year	Drug susceptibility of population	Untreated patients (N/%)	Treated patients (N/%)	Treatment duration
Riley(17)	1962	DS	67	40	Not clear
Escombe(18)	2007	Mixed*	17% of patient-days	83% of patient-days	Not clear
Escombe(19)	2008	Mixed*			Not clear
Dharmadhikari(20)	2014	MDR/XDR	Variable (across 5 studies)		Not clear

Abbreviations:
DS = drug-susceptible TB;
MDR = multi-drug resistant TB;
N = number assessed;
XDR = extensively drug resistant.

Footnotes:
* Included individuals with drug sensitive TB and those with drug resistant TB

Figures

Figure 1

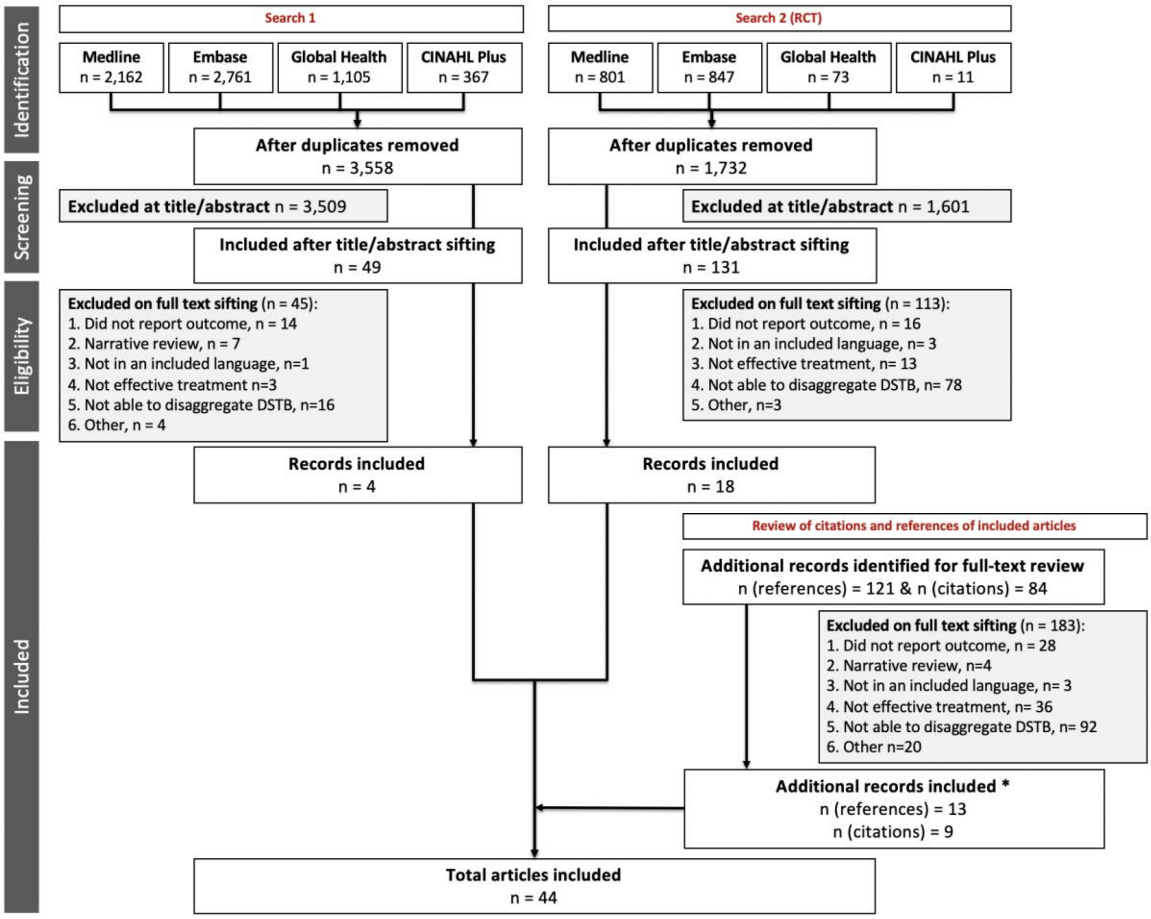

Figure 2

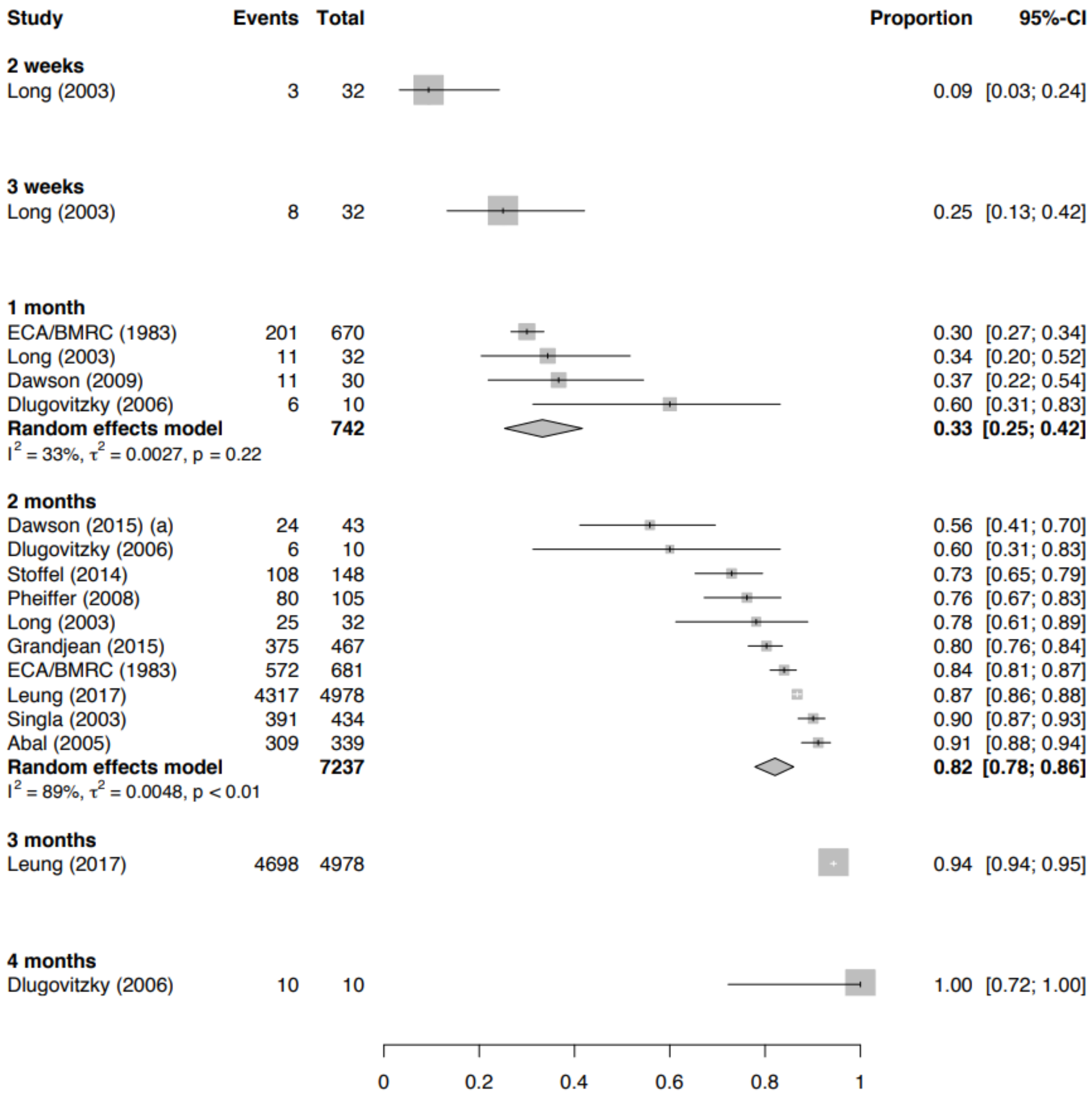
Study Events Total Proportion 95%-CI
2 weeks
Long (2003) 3 32 0.09 [0.03; 0.24]
3 weeks
Long (2003) 8 32 0.25 [0.13; 0.42]
1 month
ECA/BMRC (1983) 201 670 0.30 [0.27; 0.34]
Long (2003) 11 32 0.34 [0.20; 0.52]
Dawson (2009) 11 30 0.37 [0.22; 0.54]
Dlugovitzky (2006) 6 10 0.60 [0.31; 0.83]
Random effects model 742 0.33 [0.25; 0.42]
$I^2 = 33\%, \tau^2 = 0.0027, p = 0.22$
2 months
Dawson (2015) (a) 24 43 0.56 [0.41; 0.70]
Dlugovitzky (2006) 6 10 0.60 [0.31; 0.83]
Stoffel (2014) 108 148 0.73 [0.65; 0.79]
Pheiffer (2008) 80 105 0.76 [0.67; 0.83]
Long (2003) 25 32 0.78 [0.61; 0.89]
Grandjean (2015) 375 467 0.80 [0.76; 0.84]
ECA/BMRC (1983) 572 681 0.84 [0.81; 0.87]
Leung (2017) 4317 4978 0.87 [0.86; 0.88]
Singla (2003) 391 434 0.90 [0.87; 0.93]
Abal (2005) 309 339 0.91 [0.88; 0.94]
Random effects model 7237 0.82 [0.78; 0.86]
$I^2 = 89\%, \tau^2 = 0.0048, p < 0.01$
3 months
Leung (2017) 4698 4978 0.94 [0.94; 0.95]
4 months
Dlugovitzky (2006) 10 10 1.00 [0.72; 1.00]
0 0.2 0.4 0.6 0.8 1

Figure 3

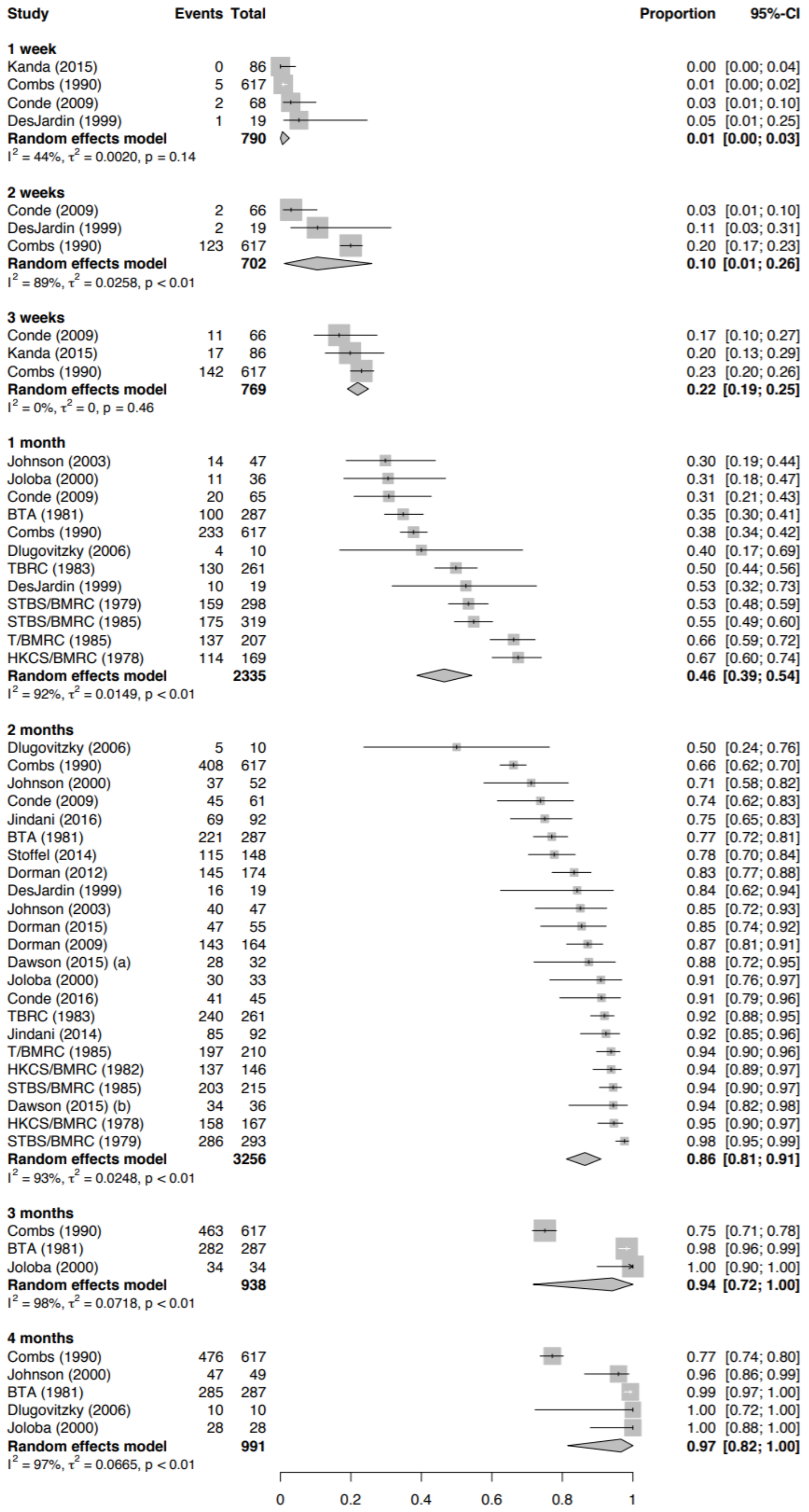

Study Events Total Proportion 95%-CI
1 week
Kanda (2015) 0 86 0.00 [0.00; 0.04]
Combs (1990) 5 617 0.01 [0.00; 0.02]
Conde (2009) 2 68 0.03 [0.01; 0.10]
DesJardin (1999) 1 19 0.05 [0.01; 0.25]
Random effects model 790 0.01 [0.00; 0.03]
I^2 = 44%, τ^2 = 0.0020, p = 0.14
2 weeks
Conde (2009) 2 66 0.03 [0.01; 0.10]
DesJardin (1999) 2 19 0.11 [0.03; 0.31]
Combs (1990) 123 617 0.20 [0.17; 0.23]
Random effects model 702 0.10 [0.01; 0.26]
I^2 = 89%, τ^2 = 0.0258, p < 0.01
3 weeks
Conde (2009) 11 66 0.17 [0.10; 0.27]
Kanda (2015) 17 86 0.20 [0.13; 0.29]
Combs (1990) 142 617 0.23 [0.20; 0.26]
Random effects model 769 0.22 [0.19; 0.25]
I^2 = 0%, τ^2 = 0, p = 0.46
1 month
Johnson (2003) 14 47 0.30 [0.19; 0.44]
Joloba (2000) 11 36 0.31 [0.18; 0.47]
Conde (2009) 20 65 0.31 [0.21; 0.43]
BTA (1981) 100 287 0.35 [0.30; 0.41]
Combs (1990) 233 617 0.38 [0.34; 0.42]
Dlugovitzky (2006) 4 10 0.40 [0.17; 0.69]
TBRC (1983) 130 261 0.50 [0.44; 0.56]
DesJardin (1999) 10 19 0.53 [0.32; 0.73]
STBS/BMRC (1979) 159 298 0.53 [0.48; 0.59]
STBS/BMRC (1985) 175 319 0.55 [0.49; 0.60]
T/BMRC (1985) 137 207 0.66 [0.59; 0.72]
HKCS/BMRC (1978) 114 169 0.67 [0.60; 0.74]
Random effects model 2335 0.46 [0.39; 0.54]
I^2 = 92%, τ^2 = 0.0149, p < 0.01
2 months
Dlugovitzky (2006) 5 10 0.50 [0.24; 0.76]
Combs (1990) 408 617 0.66 [0.62; 0.70]
Johnson (2000) 37 52 0.71 [0.58; 0.82]
Conde (2009) 45 61 0.74 [0.62; 0.83]
Jindani (2016) 69 92 0.75 [0.65; 0.83]
BTA (1981) 221 287 0.77 [0.72; 0.81]
Stoffel (2014) 115 148 0.78 [0.70; 0.84]
Dorman (2012) 145 174 0.83 [0.77; 0.88]
DesJardin (1999) 16 19 0.84 [0.62; 0.94]
Johnson (2003) 40 47 0.85 [0.72; 0.93]
Dorman (2015) 47 55 0.85 [0.74; 0.92]
Dorman (2009) 143 164 0.87 [0.81; 0.91]
Dawson (2015) (a) 28 32 0.88 [0.72; 0.95]
Joloba (2000) 30 33 0.91 [0.76; 0.97]
Conde (2016) 41 45 0.91 [0.79; 0.96]
TBRC (1983) 240 261 0.92 [0.88; 0.95]
Jindani (2014) 85 92 0.92 [0.85; 0.96]
T/BMRC (1985) 197 210 0.94 [0.90; 0.96]
HKCS/BMRC (1982) 137 146 0.94 [0.89; 0.97]
STBS/BMRC (1985) 203 215 0.94 [0.90; 0.97]
Dawson (2015) (b) 34 36 0.94 [0.82; 0.98]
HKCS/BMRC (1978) 158 167 0.95 [0.90; 0.97]
STBS/BMRC (1979) 286 293 0.98 [0.95; 0.99]
Random effects model 3256 0.86 [0.81; 0.91]
I^2 = 93%, τ^2 = 0.0248, p < 0.01
3 months
Combs (1990) 463 617 0.75 [0.71; 0.78]
BTA (1981) 282 287 0.98 [0.96; 0.99]
Joloba (2000) 34 34 1.00 [0.90; 1.00]
Random effects model 938 0.94 [0.72; 1.00]
I^2 = 98%, τ^2 = 0.0718, p < 0.01
4 months
Combs (1990) 476 617 0.77 [0.74; 0.80]
Johnson (2000) 47 49 0.96 [0.86; 0.99]
BTA (1981) 285 287 0.99 [0.97; 1.00]
Dlugovitzky (2006) 10 10 1.00 [0.72; 1.00]
Joloba (2000) 28 28 1.00 [0.88; 1.00]
Random effects model 991 0.97 [0.82; 1.00]
I^2 = 97%, τ^2 = 0.0665, p < 0.01
0 0.2 0.4 0.6 0.8 1

Figure 4

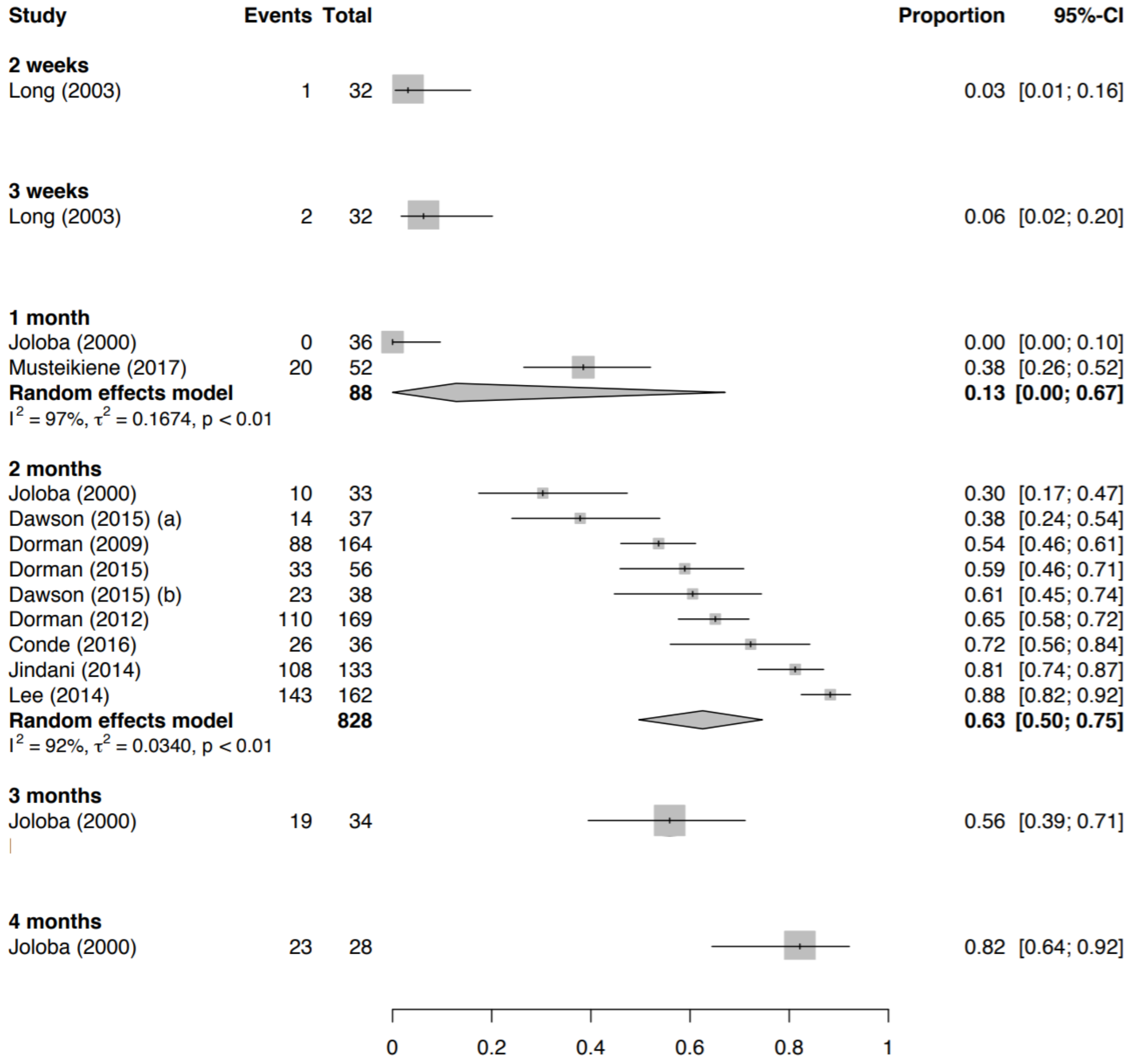
Study Events Total Proportion 95%-CI
2 weeks
Long (2003) 1 32 0.03 [0.01; 0.16]
3 weeks
Long (2003) 2 32 0.06 [0.02; 0.20]
1 month
Joloba (2000) 0 36 0.00 [0.00; 0.10]
Musteikiene (2017) 20 52 0.38 [0.26; 0.52]
Random effects model 88 0.13 [0.00; 0.67]
$I^2 = 97\%, \tau^2 = 0.1674, p < 0.01$
2 months
Joloba (2000) 10 33 0.30 [0.17; 0.47]
Dawson (2015) (a) 14 37 0.38 [0.24; 0.54]
Dorman (2009) 88 164 0.54 [0.46; 0.61]
Dorman (2015) 33 56 0.59 [0.46; 0.71]
Dawson (2015) (b) 23 38 0.61 [0.45; 0.74]
Dorman (2012) 110 169 0.65 [0.58; 0.72]
Conde (2016) 26 36 0.72 [0.56; 0.84]
Jindani (2014) 108 133 0.81 [0.74; 0.87]
Lee (2014) 143 162 0.88 [0.82; 0.92]
Random effects model 828 0.63 [0.50; 0.75]
$I^2 = 92\%, \tau^2 = 0.0340, p < 0.01$
3 months
Joloba (2000) 19 34 0.56 [0.39; 0.71]
4 months
Joloba (2000) 23 28 0.82 [0.64; 0.92]
0
0.2
0.4
0.6
0.8
1

Figure 5

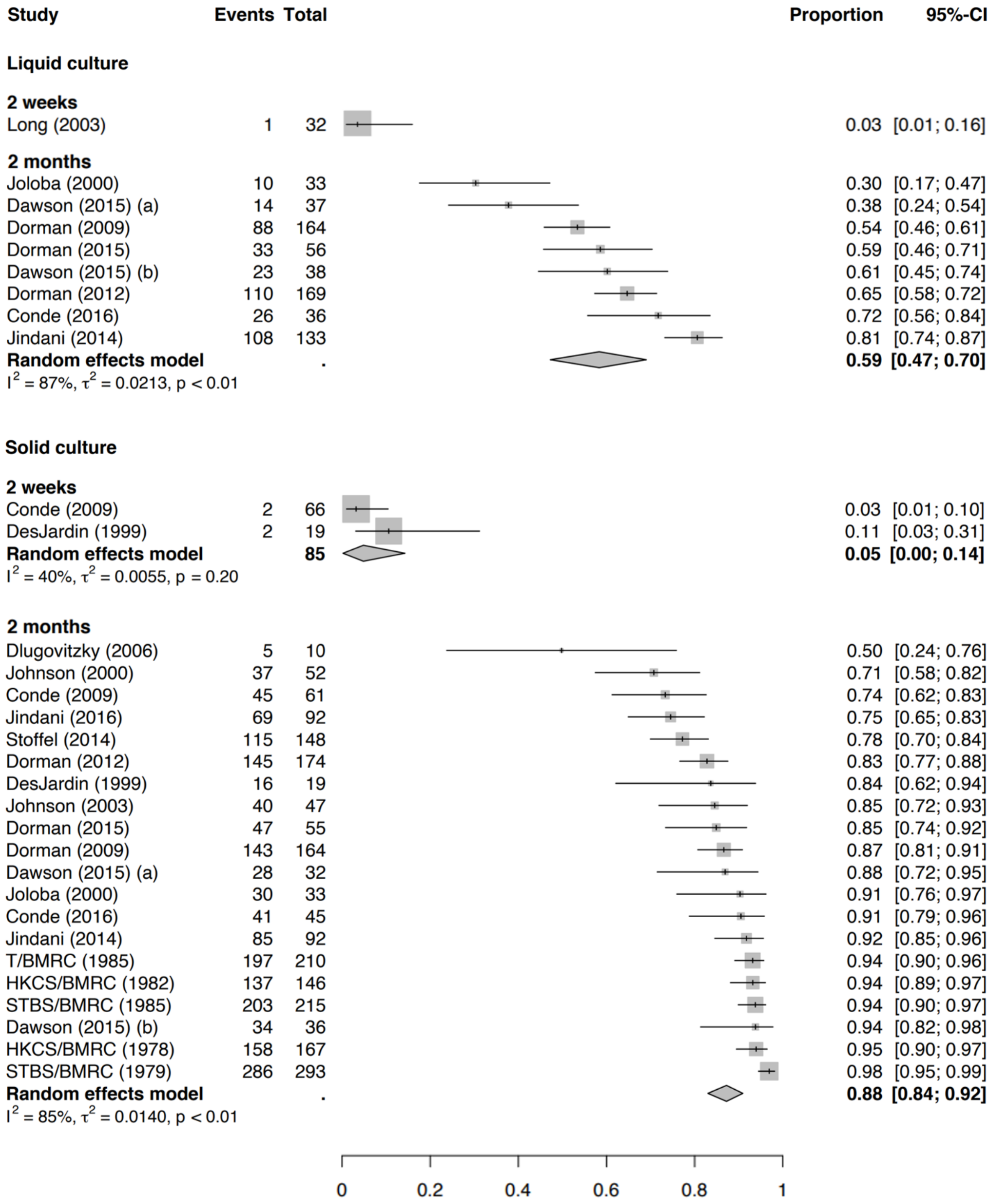
Study Events Total Proportion 95%-CI
Liquid culture
2 weeks
Long (2003) 1 32 0.03 [0.01; 0.16]
2 months
Joloba (2000) 10 33 0.30 [0.17; 0.47]
Dawson (2015) (a) 14 37 0.38 [0.24; 0.54]
Dorman (2009) 88 164 0.54 [0.46; 0.61]
Dorman (2015) 33 56 0.59 [0.46; 0.71]
Dawson (2015) (b) 23 38 0.61 [0.45; 0.74]
Dorman (2012) 110 169 0.65 [0.58; 0.72]
Conde (2016) 26 36 0.72 [0.56; 0.84]
Jindani (2014) 108 133 0.81 [0.74; 0.87]
Random effects model . 0.59 [0.47; 0.70]
I^2 = 87%, τ^2 = 0.0213, p < 0.01
Solid culture
2 weeks
Conde (2009) 2 66 0.03 [0.01; 0.10]
DesJardin (1999) 2 19 0.11 [0.03; 0.31]
Random effects model 85 0.05 [0.00; 0.14]
I^2 = 40%, τ^2 = 0.0055, p = 0.20
2 months
Dlugovitzky (2006) 5 10 0.50 [0.24; 0.76]
Johnson (2000) 37 52 0.71 [0.58; 0.82]
Conde (2009) 45 61 0.74 [0.62; 0.83]
Jindani (2016) 69 92 0.75 [0.65; 0.83]
Stoffel (2014) 115 148 0.78 [0.70; 0.84]
Dorman (2012) 145 174 0.83 [0.77; 0.88]
DesJardin (1999) 16 19 0.84 [0.62; 0.94]
Johnson (2003) 40 47 0.85 [0.72; 0.93]
Dorman (2015) 47 55 0.85 [0.74; 0.92]
Dorman (2009) 143 164 0.87 [0.81; 0.91]
Dawson (2015) (a) 28 32 0.88 [0.72; 0.95]
Joloba (2000) 30 33 0.91 [0.76; 0.97]
Conde (2016) 41 45 0.91 [0.79; 0.96]
Jindani (2014) 85 92 0.92 [0.85; 0.96]
T/BMRC (1985) 197 210 0.94 [0.90; 0.96]
HKCS/BMRC (1982) 137 146 0.94 [0.89; 0.97]
STBS/BMRC (1985) 203 215 0.94 [0.90; 0.97]
Dawson (2015) (b) 34 36 0.94 [0.82; 0.98]
HKCS/BMRC (1978) 158 167 0.95 [0.90; 0.97]
STBS/BMRC (1979) 286 293 0.98 [0.95; 0.99]
Random effects model . 0.88 [0.84; 0.92]
I^2 = 85%, τ^2 = 0.0140, p < 0.01
0 0.2 0.4 0.6 0.8 1

Figure 6

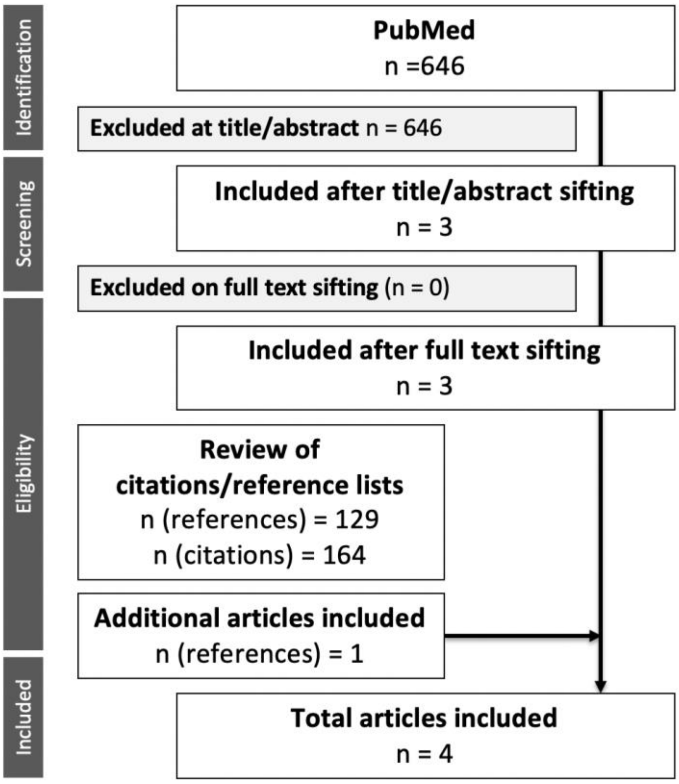
Identification
Screening
Eligibility
Included
PubMed
n =646
Excluded at title/abstract n = 646
Included after title/abstract sifting
n = 3
Excluded on full text sifting (n = 0)
Included after full text sifting
n = 3
Review of
citations/reference lists
n (references) = 129
n (citations) = 164
Additional articles included
n (references) = 1
Total articles included
n = 4
