# Supplementary material for: Dynamics of sputum conversion during effective tuberculosis treatment: A systematic review and meta-analysis
Source: PLoS Med. 2021 Apr 26;18(4):e1003566. doi: 10.1371/journal.pmed.1003566 (PMC8109831; doi:10.1371/journal.pmed.1003566)
Supplement: Attachment — Submitted filename: Rebuttal letter.docx [file pmed.1003566.s012.docx]

**Dr Emma Veitch PhD**

**PLOS Medicine**

**6^th^ October 2020**

**Revision letter - PMEDICINE-D-20-03202R1**

**Dynamics of sputum conversion during effective tuberculosis treatment: a systematic review and meta-analysis**

Dear Dr Veitch,

We write in response to your email of 16^th^ September 2020 and thank you for the insightful feedback from the editors and reviewers. We have given careful consideration to all comments and queries and here present our responses. All revisions to the original manuscript are highlighted in the attached ‘marked up’ version and are referenced below by page and line number highlighted in yellow.

We acknowledge that the manuscript has been strengthened and improved by these amendments and are grateful to the reviewers for their role in this process. We look forward to hearing from you again soon.

Yours sincerely, on behalf of the authors

**Claire Calderwood**

**James Wilson**

**Dave Moore**

**Professor of Infectious Diseases and Tropical Medicine**

**Requests from the editor**

*Please structure your abstract using the PLOS Medicine headings (Background, Methods and Findings, Conclusions).

- We have restructured the abstract

*We recommend ensuring that the abstract (ideally Methods and Findings section) includes a brief note about any key limitations of the study's methodology.

- We have added a comment about limitations of our methodology and results [PAGE 2, LINES 36-38 and PAGE 3, LINES 45-47]

*At this stage, we ask that you include a short, non-technical Author Summary of your research to make findings accessible to a wide audience that includes both scientists and non-scientists. The Author Summary should immediately follow the Abstract in your revised manuscript. This text is subject to editorial change and should be distinct from the scientific abstract. Please see our author guidelines for more information: <https://journals.plos.org/plosmedicine/s/revising-your-manuscript#loc-author-summary>

- We have added an author summary [PAGES 4/5, LINES 54-79]

*The methods section of the paper notes that the systematic review was conducted per PRISMA guidelines, in general PRISMA is described (or was conceived) as a tool to assist reporting, not necessarily conduct - it might be an idea to rephrase this as the SR being reported per PRISMA (rather than conducted). We'd also suggest including a completed PRISMA checklist as supporting information alongside the paper.

- Thank you. We have updated our wording to reflect this [PAGE 7. LINES 117-119]
- We have included the completed and updated PRISMA checklist – [S6 Table]

*The discussion section at the moment does not seem to include much of an explicit acknowledgement of main limitations of the SR methods (or evidence base), some issues relating to generalisability and clinical implications are discussed but not really "signposted" as limitations of the SR per se. We'd recommend adding something to more explicitly describe this.

- Thank you for this helpful comment, we have re-worded these sections of the discussion to more explicitly show the limitations of the systematic review [PAGE 27, LINES 412-420; PAGE 28, LINE 441; PAGE 29, LINES 448-9]

*One reviewer queries the definition of what data to include in the SR - the editors (including academic editor) acknowledged that it would not be possible to entirely address this issue without totally redoing the SR, so we'd just recommend including a discussion on this point in relation to study limitations (if any).

- We have discussed this further in the response to the comments made by the reviewers below, which we hope gives additional clarity on our decisions around the scope of the analyses included in the review. We have also highlighted this as a limitation of the review in the discussion [PAGE 27, LINES 412-416].

**Reviewer #1:**

This review looked at culture conversion in smear positive pulmonary tuberculosis patients after several durations of effective anti tuberculosis therapy.

The findings indicate that the majority of patients (97% by liquid culture) are still culture positive after two weeks of effective treatment.

These findings are of great interest to infection control policy makers.

I have several questions for the authors to consider

1. Would it be possible to do a subgroup analysis or HIV infected vs HIV uninfected patients?

This is an important question which we considered carefully when writing the original manuscript. Ultimately, we decided that the answer to this question was “no”, for the reasons we lay out here.

Few studies presented analyses stratified by HIV status; two report data for time to conversion among patients with drug-sensitive TB receiving effective treatment (summarised below) and none presented proportion converted data in the same population. Dominguez-Castellano et al. highlight the presence of other differences in TB disease, such as the presence of cavitation, between patients with and without HIV and, without the further ability to explore these differences through meta-regression, we questioned how much additional information was added by these two studies.

An alternative approach could have been to compare studies that excluded people living with HIV to those which included them (at a variable proportion of the study population). The highly variable proportion of patients with HIV amongst ‘mixed’ studies renders this approach unhelpful; furthermore a number of studies excluded patients with advanced disease (e.g. Conde 2009, Combs 1990, Dawson 2015 a and b, Dorman 2015, Jindani 2014).

As a consequence we did not explore this further in the main analysis and this explanation underlies our comment in the discussion that “Lack of disaggregation by conversion-predicting covariates and other key patient characteristics, such as HIV and the presence of cavitating lung disease, prevented any meaningful attempt to analyse the heterogeneity between and within subgroups” [PAGE 28, LINES 433-436].

By way of example, to illustrate how disentangling the data ends up with meaningless metrics that don’t get to the heart of the question:

- *Kennedy 1996: report time to first negative sputum smear or culture (method not-specified) result, stratified by HIV status (32 HIV+ and 54 HIV- patients). Median time to smear conversion was two months among PLHIV (range 1-4 months) and 1 month among those without HIV (range 1-3 months). Median time to culture conversion (would not have been included in our main analysis as of unspecified method) was 2 months among PLHIV (1-4 months) and 2 months among those without HIV (1-3 months).*
- *Dominguez-Castellano: report time to smear conversion, stratified by HIV status (42 HIV+ 67 HIV-), finding that the median number of days to smear conversion was shorter in PLHIV (18 ± 2 days [1SE] vs 30 ± 4 days; p=0.011) however 9.6% of those with HIV had presence of lung cavitation, compared to 52.2% of those without HIV.*

2. Was there any difference in culture conversion time between patients receiving and not receiving ethambutol?

On reviewing Table 2; there are six studies that included patients taking HRZ during the intensive phase that could potentially be compared with data from equivalent HRZE arms: Abal 2005, Chaulet 1995, Long 2003, STS/BMRC 1985, Coombs 1990 and Leung 2017. Leung 2017 and Coombs 1990 do not disaggregate by whether ethambutol/streptomycin were included, leaving us with:

Chaulet 1995:	250 patients on RHZ with 2 month culture (culture media not specified)

Long 2003:	7 patients on RHZ with 2 weekly smears

Abal 2005:	11 patients on RHZ with 2 month smear

STS/BMRC 1985:	113 patients on HRZ with 1,2,3 month liquid culture

Given that we are not presenting data where culture media is unknown (significant observed difference between liquid and solid culture media conversion times), and given the very low number of patients in the Long and Abal studies, this essentially leaves us with only the STS/BMRC 1985 study to compare with equivalent HRZE arms for liquid culture media. We believe that this analysis would not be helpful given multiple other systemic differences defined by being in the STS/BMRC study, eg study type, year, location, severity of illness.

3. Was any consideration given to looking at culture conversion times in smear negative culture positive patients?

Yes; although only two studies disaggregated data by baseline smear status:

BTA 1981: RCT looking at solid culture conversion at 1, 2 and 3 months

Lee 2014: Retrospective cohort study looking at liquid culture conversion at 2 months

Within these studies, the number of baseline smear-negative patients was small. Given only 2 studies that disaggregate by baseline smear status, and looking at different culture methods, it was felt this subgroup analysis would not be helpful given other confounders at play. Alternately, a ‘mixed baseline smear status’ vs ‘positive baseline smear status’ analysis could be performed, as with HIV status, but again this was felt to be of very limited use.

We have added “baseline smear status” to the list of covariates where meaningful subgroup analysis was not felt to be helpful due to marked paucity of disaggregated data. [PAGE 28, LINE 434]

4. Was it possible to look at the effect of smear grade or initial time to culture on culture conversion at 2 weeks?

Two studies provide data stratified by smear grade in the population of interest, one of which also looked at initial time to culture.

Musteikiene 2017: liquid culture conversion at one month of treatment by smear grade <2+ vs ≥2+. 71% of those with smear grade <2+ culture converted (N=21) compared to 16.1% of those with grade ≥2+ (N=31). Similarly, by initial time to positive culture <10 days, 20% converted by one month (N=20) compared to 50% of those with ≥10 days to initial positive culture (N=32).

Dominguez-Castellano 2003: time to smear conversion (TTC) comparing individuals with ‘scarce’ (N=30) vs ‘abundant’ (N=79) AFB, however these terms were not defined in terms of smear grade. For individuals with scare AFB, TTC was 12 days (SE±0) compared to 30 days for those with abundant AFB. (SE±3) (P<0.001).

As before, given the small numbers of participants, in only two studies for which data could not be combined and definitions lacked clarity, each with specific study population profiles with distinct disease severity, comorbidity and demographic profiles, we felt that inclusion and specific mention of these individual findings would present a misleading picture and afford them greater prominence than was warranted.

**Reviewer #2:**

I confine my remarks to statistical aspects of this paper. These were well done and I recommend publication.

We are grateful for the statistical review and the generous remarks.

**Reviewer #3**

This is a well-written paper, with a clear research question and appropriate methodology and conclusions. The topic is of significant clinical relevance, and would be a significant addition to the current knowledge. Regarding statistics I do not have relevant experience to assess the accuracy.

We are grateful for the review and the observation that this work advances knowledge.

**Reviewer #4**

Summary

This is a systematic review commissioned by WHO TB GDG with the aim of summarising how long MTB can be detected in sputum in population of adults initiating treatment for pulmonary tuberculosis, as a proxy for "potential infectiousness". The authors look at 2 summary measure types (time to conversion from positive to negative, and proportion converting to negative at given timepoints) for two method types (microscopy smear and culture). In addition, a narrative review of animal studies is included. Unsurprisingly the authors find that a majority of PTB patients remain smear and culture positive after 2 weeks treatment, but with substantial heterogeneity between studies in all outcomes. The authors found that the literature is not well enough reported (ie disaggregated) to allow any quantitative analysis of this heterogeneity (eg meta-regression). They report that the animal study data does not provide any estimates of infectiousness change over time on treatment and hint at suspected bias from incomplete data (probably there is more detail given in the supplement). The authors conclude that "potential infectiousness" extends well into TB treatment, but are careful to note that "What this means for TB transmission and the two-week 'rule' cannot be determined by these analyses".

Comments

The review is very well written, and meets its objectives using sound analyses which are well presented.

It meets all PRISMA 2009 checklist criteria (assume PRISMA item 8 & 11 are in the supplement), except there is no mention of systematic review registration number or indication if a review protocol exists / if/ where it can be accessed (PRISMA 2 & 5).

Thank you for bringing this to our attention. We have included the PRISMA checklist in the supplementary material [S6 Table], and referenced the prospectively registered review protocol in the abstract [PAGE 3, LINE 52] and methodology [PAGE 7, LINE 117]. PRISMA 8 and 11 are available in the supplementary material [S1-3 Tables] and [S1 Form] respectively.

The lit search was performed 2.5 years ago.

Any possible major weaknesses stem from the terms of reference for the review (commissioned by WHO) rather than the manuscript itself I think. E.g. the two week rule comes from a review by Rouillon in Tubercle 1976 and was based on the absence of reported cases of TST conversion in household contacts of PTB patients after the index patients were discharged on treatment (which ignores the survivor bias from these contacts likely having already been exposed and not converted). I wondered why the animal data was reviewed but not this type of epidemiological data. I also thought the EBA pharmacodynamics based on serial quantification of bacilli in sputum could have been relevant to the review question (not least because it is much better powered to assess the heterogeneity issue). However as stated above the review is very clear and meets the stated objectives completely so this is just an aside.

Thank you for these insightful comments. We did discuss amongst ourselves the merits of including EBA data and TST conversion data in humans even though, as the reviewer correctly notes, these were outside the WHO commission remit. EBA studies (typically phase 2 trials) often only report the mean log change CFU from 0-2 days, and not a proportion converted – which does not directly address conversion. Furthermore, drawing on TST conversion data in animals was felt potentially more useful than human TST conversion data given the controlled conditions used in the animal studies, and the issue of survivor bias in the human studies mentioned by the reviewer.

We have included a statement in the abstract and introduction clarifying that these data were outside the scope of the review. [PAGE 2, LINES 36-38; PAGE 6, LINES 112-114]

Minor comments

Typo in table 1:

Any study in a not reporting data on participants with confirmed, drug susceptible…

Thank you for highlighting. This has been corrected [PAGE 8, TABLE 1]

Line 167: Thirty-six studies reported data on culture conversion; 9 using liquid culture and 23 using solid (8 studies reported both).

I had to read this a few times to understand why couldn't make 36 from 9, 23, and 8 - it only makes sense when you read the next line. Would be clearer if edited maybe.

We have simplified – thank you for noting. [PAGE 20, LINES 293-5]

**Reviewer #5**

The authors did a great work in analysing the sputum conversion time after treatment onset. The study design, meta-analyses, and write-up of the study is well done.

However, I am quite worried about the conclusion, due to the smaller sample size obtained from the included studies.

Thank you for highlighting this important point. We recognise that many of the identified studies include a relatively small number of patients, contributing to both between-study heterogeneity and statistical uncertainty. We have added a sentence that addresses this, and we highlight the 95% confidence intervals that account for this. [PAGE 27, LINES 418-420].

I am also worried about the inclusion of the animal components when it did not add up anything to the conclusion or to strengthen the study.

We agree that the combined analysis of studies reviewing human-to-animal transmission does not provide any additional evidence to enhance our understanding of sputum conversion dynamics. We believe it is nonetheless important to include this ‘negative result’ in the review given that (i) it was our intention from the outset (and an explicit request of the WHO GDG) to perform this review, and (ii) to our knowledge this is the first time evidence of this form has been combined and systemically reviewed. We believe the readers would be interested to note that such a review has been undertaken regardless of the outcome.

We recognise this outcome in the discussion [PAGE 29, LINES 457-460 – no new edits maed]. The full narrative review is included in the supplementary material [S1 Appendix].

Other concerns and comments are found in the included/attached PDF.

*Re: Lines 15-16 ”conversion in 9% of patients at two weeks and 82% at two months of treatment (95% confidence interval (CI), 3–24% and 78–86%, study numbers (N) = 1 and 10)”:*

I am sure the CI values are respective to the 9% and 82%? Yet there is nothing in the sentence to suggest this, but the reader is left to guess and figure this out.

Further, what does the study numbers of 1 and 10 stand for? Does the 1 represent the 9% and the 10, 82%? This is very confusing. The authors must endeavour to rewrite this to make it clearer and unequivocal for the reader

Thank you for highlighting this. We have re-written this to make it clear to which estimates the CIs and number of contributing studies relate. [PAGE 2, LINES 40-1]

*Re: Lines 21 -22 “Most patients remained culture-positive at two weeks, challenging the view that individuals are not infectious after this interval.”*

I am particularly concerned about this conclusion, because the number of studies/patients used to make this conclusion seems relatively small to me. I am doubtful such a small number can be used to make a general conclusion.

This seems preliminary and might require more studies to confirm it. Particularly, the number of studies & patients may need to be increased to strengthen the conclusion.

We acknowledge the comment though similarly acknowledge that the view that individuals are not infectious is not supported by any data. We address this in our earlier response to the comment about small study numbers above. The statistical uncertainty resulting from the small numbers is directly reflected in the confidence intervals. For smear-positive patients, the 2-week estimate for solid culture conversion is 5% (95% CI 0 – 14%) and for liquid culture conversion is 3% (95% CI 1-16%); we believe our conclusion that ‘the majority remain culture-positive at two weeks’ is a fair interpretation of these estimates.

*Re: Line 34 “National guidelines”*

Which nation is being referred to here? The authors are from two countries, SA and the UK, so which are they referring to? Please be specific as this is an international journal.

Thank you for raising this. We are referring to UK, USA and Canadian guidelines with references [6-8]. We have (i) amended the text to make it clear we are referring to multiple countries and (ii) added a reference to the SA guidance. [PAGE 5, LINES 89-91]

*Re: presentation of numbers, multiple entries*

We have reviewed all numbers written as numerals vs text, and amended such that numbers at the beginning of sentences and numbers < 10 are written out as text [MULTIPLE LINES IN RESULTS AND DISCUSSION]

*Re: Lines 140, 154, 186 spelling/grammar suggestions*

Amended as suggested – thank you for highlighting.

*Re: Line 192 “Animal studies”*

If these animal studies could not help clarify or add up to the conclusion on the time to conversion in the infected animals, or the infectiousness of the treated patients after 2 weeks, then why add it/include it in the meta-analyses? What purpose are these studies really serving as far as the study's objectives are concerned?

*Re: Lines 211-212 “There are no data that describe how infectiousness changed over time after treatment initiation”*

Doesn't this defeat the purpose for including these studies in this review?

*Re: Lines 214-217: “It is not possible from these data to draw conclusions about the role of effective treatment in reducing infectiveness of TB, nor on the time-frame over which any reduction in infectiousness occurs”*

Very good. Yet the authors still believe it's worth including it?

Or they're convinced it's useful information, albeit negative? I am sure this is the reason and a good one?

*Re: Lines 227-228: “The evaluation of TB transmission from human patients to experimental animals provides an additional perspective”*

Yet, this could not support or take away from the conclusion of this study? What additional perspective was really provided with respect to the study's objective on sputum or culture conversion within a particular time frame?

We hope our second response above to this reviewer addresses these further comments regarding the animal studies.

*Re: Lines 234 – 239: After 2 weeks of effective treatment, 95% and 97% of baseline smear-positive patients demonstrate persistently positive solid and liquid cultures respectively. This directly challenges guidance that infection, prevention and control measures can be relaxed following 2 weeks of effective treatment. On completion of the 2 month intensive treatment phase, 12% and 41% of baseline smear-positive patients remain solid and liquid culture-positive respectively.*

I realise that the TTC for liquid cultures were longer than solid media. Could the authors provide reasons for that observation here in the discussion?

Thank you picking up on this point. As addressed in a later paragraph in the discussion [PAGE 28, LINES 428-432, no new edits], this difference is not unexpected given the increased sensitivity of liquid culture media. Unfortunately, the inability to control for both known and unknown confounders (eg HIV, adherence, cavitation, etc), and hence the heterogeneity observed between studies, really muddies the water, preventing anything more meaningful than speculation.

*Re: Lines 264-265: “… unlike those for liquid culture estimates, predated the HIV epidemic, whilst people living with HIV have been shown to have shorter culture conversion times”*

Then why did this not give the liquid cultures shorter conversion times, if most of the solid media conversion studies were probably HIV-negative?

This sentence is confusing and should be clarified further.

We agree – this sentence is confusing. We were attempting to illustrate how confounding can challenge how well the data demonstrate association between proportion converted at the 2 month time point, and the culture media used. On reflection, we have decided to remove this sentence.

*Re: Lines 297-298 “What this means for TB transmission and the two-week ‘rule’ cannot be determined by these analyses”.*

I agree. I wish the authors could also touch on the smaller sample size involved in these studies included in the meta-analyses.”

We hope that our previous comments addressing the small study sizes, and our addition to the discussion provides sufficient recognition of this point.
