# Supplementary material for: Dynamics of sputum conversion during effective tuberculosis treatment: A systematic review and meta-analysis
Source: PLoS Med. 2021 Apr 26;18(4):e1003566. doi: 10.1371/journal.pmed.1003566 (PMC8109831; doi:10.1371/journal.pmed.1003566)
Supplement: Attachment — Submitted filename: response to reviewer_new.docx [file pmed.1003566.s013.docx]

**Response to reviewers**

December 4^th^ 2020

**Request 1:** Thank you for providing your PRISMA checklist. Please replace the page numbers with paragraph numbers per section (e.g. "Methods, paragraph 1"), since the page numbers of the final published paper may be different from the page numbers in the current manuscript.

Page numbers have been replaced with paragraph numbers per section in the attached PRISMA checklist, thank you for this insight.

**Request 2:** Data statement: Is it more accurate to say “Data are available from the primary studies, all of which are published.”

This has been updated. Thank you for highlighting.

**Request 3:** In the Abstract Methods and Findings, please provide the databases searched, the beginning and end dates of your search, types of study designs included, eligibility criteria, and synthesis/appraisal methods.

We hope we have addressed these points as below:

- Line 31 and 42: we have added details of the databases search, beginning and end dates.
- Line 34 and 38: we have added further details of eligibility criteria and study types included
- Line 40 states the synthesis/appraisal methods

**Request 4:** Please begin the Abstract Conclusions with “This systematic review found that most patients remained…”.

Thank you, we have made this change as below:

- Line 56: altered start of sentence in the form “this study found that”
- Line 57: added “of TB treatment”

**Request 5:** The last sentence of the Abstract Conclusions should be replaced or modified to suggest what is needed to understand the other components of infectiousness and integrate the findings of this study.

Thank you. We have expanded this (line 58) to suggest what is needed to better understand infectiousness and integrate study findings.

**Request 6**: Author summary, line 58: instead of “not strong”, perhaps “challenged”?

Thank you for this suggestion, we have changed this (line 73).

**Request 7:** The searches are nearly 3 years old, please update to the present time.

We thank the reviewers for raising this important point which we have carefully considered this as discussed in the covering letter. We have added additional information in S2 Appendix.

**Request 8:** Discussion, line 388, please remove “the best”.

We have done this.

**Request 9:** Please present and organize the Discussion as follows: a short, clear summary of the article's findings; what the study adds to existing research and where and why the results may differ from previous research; strengths and limitations of the study; implications and next steps for research, clinical practice, and/or public policy; one-paragraph conclusion.

We have revisited the discussion in detail and made changes to structure as requested by the editors and more clearly highlight the aspects of our study we consider to be strengths and weaknesses, and how this study fits into the wider literature. Details of these changes are:

- Line 36-397 and 408: changed ‘tuberculosis’ to ‘TB’ and ‘infection control’ to ‘IPC’ where applicable for consistency within the manuscript (not requested by editors)
- Line 401: changed ‘optimal’ to ‘effective’
- Line 406: paragraph starting “This review draws together” moved down so summary of article’s findings (starting line 408) becomes the first section of the discussion.
- Line 416: new paragraph inserted highlighting first what this study adds to existing research and second, strengths of the study, including incorporating the paragraph starting “This review draws together” that has been moved down.
- Line 422: paragraph substantially altered to frame these limitations of the study better in the context of the existing research. We hope this adds context and clarity. The original version of this paragraph is included here as an endnote.^[1]^
- Line 437: paragraph reworked to more clearly highlight how these aspects are limitations of the study. Reference to confounding moved to later paragraph as below.
- Line 451: changed from ‘perhaps expected’ to ‘unsurprising’ (not requested by editors)
- Line 454: removed the phrase ‘and other key patient characteristics’. Whilst other characteristics not previously explored may influence sputum conversion dynamics, the discussion of potential confounding is made clearer by restricting this to established conversion-predicting covariates.
- Line 456: changed from ‘analyse the heterogeneity’ to ‘explore potential confounding’
- Line 456: removed ‘additionally, [point estimates]’
- Line 457: changed from ‘a further parameter that was infrequently reported’ to ‘however this parameter was often not reported’.
- Line 458: added ‘These limitations should be recognised when inferring a relationship between detection method and conversion estimates’ – a rewording of the previous sentence moved from the preceding paragraph: ‘when inferring a relationship between detection method and conversion estimates this often nuanced confounding should be recognised’.
- Line 460: Reframed discussion of the review’s scope with regard to drug-susceptibility for clarity as a limitation.
- Line 467: minor changes to first sentence to remove unnecessary words – from ‘adherence to therapy is a critical component of effective treatment’ to ‘adherence to therapy is critical to effective treatment’ – and divided into two sentences (removing colon).
- Line 488: reframed the second part of this paragraph to build on public health implications (line 610) and discuss next steps for research to address these gaps.
- Line 495: new one paragraph conclusion.

**Request 10**: The contents of S3 Table read “Table E3”

Thank you, this has been amended

**Request 11**: S1 Form is empty, can you provide the completed form?

Thank you for highlighting this oversight. The form is now populated with the extracted data.

**Reviewer #1:** Thank you for addressing the questions I raised in the review. These have all been answered. 

**Reviewer #5:** I thank the authors for such a good work. I am convinced this will be a good addition to science, public health and the literature. Congratulations on such a good work!

Thank you for these comments and we are grateful to you for the insightful comments you provided on the previous version, consideration of which has resulted in an improved manuscript.

1. Assessment of the capacity to detect *Mtb* in spontaneously produced sputum does not directly quantify infectiousness, which also depends on environment, cough dynamics and recipient factors. The core of this analysis relates to culture conversion as this is an assessment of presence of viable *Mtb* in respiratory samples; we included data on smear conversion as the most frequently used microbiological test used for response to therapy, however smear-positivity of treated patients may not reflect presence of viable *Mtb*.

   Presence of *Mtb* in respiratory secretions is necessary, although in the absence of a mechanism for propagation, not sufficient for infectiousness. Cough frequency diminishes significantly in the first few weeks of effective TB treatment; this likely plays an important role in reducing transmission risk.(4, 11) In addition to excluding studies investigating mode of transmission, we also excluded studies reporting human TST conversion and quantitative measures of mycobacterial load in response to treatment, such as early bactericidal activity studies. The restriction of this review to bacteriological clearance and animal studies potentially limits its ability to inform TB IPC policy.
